# Advancing multilevel Bayesian networks with efficient Bayesian inference

**DOI:** 10.1177/09622802261468020

**Published:** 2026-09-10

**Authors:** Bezalem Eshetu Yirdaw, Legesse Kassa Debusho, Janet van Niekerk, Håvard Rue

**Affiliations:** 1 Department of Statistics, 274782 University of South Africa, Johannesburg, South Africa; 2 Statistics Program, CEMSE, 98822 King Abdullah University of Science and Technology (KAUST), Thuwal, Kingdom of Saudi Arabia; 3 Department of Statistics, University of Pretoria, Pretoria, South Africa

**Keywords:** Gaussian nodes, hybrid nodes, integrated nested Laplace approximation, LKJ prior, multilevel Bayesian network, maximum likelihood estimation, prior sensitivity, Wishart prior

## Abstract

Bayesian networks (BNs) are widely used tools for the modeling of complex dependencies among variables. However, their application to multilevel or clustered data remains constrained, especially in scenarios involving multiple random effects, due to a lack of methods and more pressing, efficient computational frameworks. In this article, we propose an innovative framework, INLA-MBN, that integrates multilevel BNs (MBNs) with the integrated nested Laplace approximation (INLA), thereby facilitating efficient structure and parameter learning in both longitudinal and cross-sectional multilevel data contexts. To the best of our knowledge, this is the first implementation of INLA for BNs that involves more than one correlated random effect. More precisely, we advance existing research by modeling longitudinal data with two correlated random effects and multilevel cross-sectional data with more than two correlated random effects. Our approach encompasses both Gaussian and hybrid MBNs that incorporate Gaussian and categorical nodes. Through an exhaustive simulation study, we demonstrate that INLA-MBN exhibit superior performance compared to networks based on maximum likelihood estimation, particularly, in scenarios characterized by small group sizes or limited repeated measurements per subject. INLA-MBN achieves a fully inferred MBN, while negating the cost associated with MBNs based on Markov Chain Monte Carlo. The proposed method was also applied to real-life longitudinal child morbidity data to demonstrate its practical applicability.

## 1. Introduction

A Bayesian network (BN) is a statistical construct for modeling intricate dependencies and estimating conditional probabilities among multiple response variables.^1–3^ This method has been applied in various fields of study, including healthcare research.^1,4,5^ For example, an earlier study^1^ investigated predictors of childhood diarrhea using a score-based causal BN. Another study^6^ also used these methods as a prediction model for multiple chronic diseases. However, data featuring a hierarchical or multilevel structure frequently appear in health science research and other fields, potentially violating the assumption of independent observations. A multilevel model is a robust choice when the independent observation assumption is disrupted, offering a solid framework to model hierarchical or multilevel data.^7,8^ Although the multilevel regression model is widely used as a statistical approach to manage correlations arising from clustering, it imposes a limitation when examining the interdependencies of multiple outcomes simultaneously. In addition, unlike BNs, it requires the outcome variable to be specified in advance. To mitigate this limitation, previous studies^9,10^ combined multilevel modeling with a BN to account for the hierarchical or multilevel structure of the data during the structure and parameter inference of the BN. These studies used a connected three-parent set block Gibbs sampler with the Bayesian information criterion (BIC) score to infer the structure of the directed acyclic graph (DAG), and maximum likelihood estimation (MLE) to estimate the parameters of the multilevel BN (MBN). Furthermore, a previous study adopted a BIC score from a mixed-effects model for each local network to learn the structure of BNs for correlated datasets.^11^ Notably, the study shows that the performance of this approach decreases for data with a small number of groups and a small number of observations per group. This may be due to the limited performance of the MLE method used in the mixed-effects models of each local network during the structure learning of the BNs. For instance, various studies revealed that the robustness of mixed-effects models is affected by the number of groups and group size.^12–19^ Furthermore, we also observed that the performance of learning an MBN using the BIC score from a mixed-effects model for each local network is highly influenced by both the group size and the number of groups.

Bayesian method enables the incorporation of prior knowledge, thereby reducing sample size bias when fitting models to small datasets while also quantifying the associated uncertainty. Among the various Bayesian computational techniques, the Markov Chain Monte Carlo (MCMC) method is a widely used approach with its own set of strengths and limitations, most notably a very high computational cost for complex models. The integrated nested Laplace approximations (INLA) methodology has emerged over the past decades as a powerful and efficient approach for Bayesian inference, particularly in models with complex random effect structures.^20,21^ Unlike the traditional MCMC methods, INLA offers fast and accurate approximations to posterior distributions, making it highly suitable for computationally intensive models.^20^ Earlier studies have demonstrated that INLA performs accurately at a lower computational cost than MCMC.^22–24^ INLA has been widely applied across various fields, including spatial modeling,^25–27^ joint modeling of longitudinal and survival outcomes,^28^ and several other complex modeling scenarios. Its flexibility also makes it an excellent choice for analyzing multilevel and longitudinal data,^29^ where correlated random effects are commonly encountered. INLA has been continuously improved to enhance computational efficiency and reduce the cost of Bayesian model estimation,^21^ making it particularly well-suited for hierarchical data with complex dependency structures.

The MBN can benefit significantly from the Bayesian approach in score computation and parameter estimation, especially in small sample scenarios. However, since MBNs require estimating hierarchical Bayesian models for many local networks during the network structure estimation, relying on MCMC methods can be computationally expensive. Therefore, this study proposed a novel approach, called INLA-MBN, that adopts INLA for computing the score of the local networks in the DAG of the MBN during structure inference. Following Scutari et al.,^11^ the hill climbing algorithm was used to identify the optimal DAG. Moreover, the goal of the BN model is not only to identify the complex dependencies between variables, but also to estimate the parameters of the joint and conditional probability distributions of all variables considered in the study. Depending on the network’s complexity, both structure and parameter estimation require a sufficient amount of data. In the case of large sample scenarios, the maximum likelihood (ML) method can effectively learn the structure and parameters of the network at an asymptotic rate of $\sqrt{n}$ .^11^ However, when the data has a hierarchical structure, the introduction of random effects adds additional complexity to the model, which makes the structure and parameter estimation with MLE more challenging and impractical. Therefore, following the score computation during structure inference, parameter estimation was also carried out using INLA.

The remaining sections of the article are organized as follows. Section 2 briefly describes the methodology used in this study, including an overview of MBNs and INLA-MBN, the representation of local networks as latent Gaussian models, the INLA methodology, and relevant computational details. Section 4 outlines the simulation setup and results based on longitudinal and cross-sectional multilevel data. Section 5 illustrates how the proposed model can be applied to real world data. Finally, Section 6 provides a discussion of the proposed method and further insights.

## 2. Method

### 2.1. Bayesian network

BNs are probabilistic graphical models that represent a collection of random variables and their conditional dependencies through a DAG D . Each node in D corresponds to a random variable, while edges encode stochastic dependencies. Let $Y=(Y_{1},\ldots ,Y_{P})$ represent a vector of random variables, learning BN B from the data Y involves structural discovery of the graph D and estimating model parameters within the parameter space Θ which is given by the following equation:

(1) $$P(B|Y)=P(D,\Theta |Y)=\underset{\text{Structure learning}}{\underbrace{P(D|Y)}}\cdot \underset{\text{Parameter learning}}{\underbrace{P(\Theta |D,Y)}}$$

The first step in BN is learning the structure of the graph which is given by the following equation:

(2) $$\begin{array}{l} p(D|Y)=\frac{p(Y|D)p(D)}{\sum_{D\in \Omega }p(Y|D)p(D)} \end{array}$$

where p(D) is the graph prior and p(Y|D) is the likelihood, which can be factorized into local probability distributions based on the Markov property as follows:

(3) $$\begin{array}{l} p(Y|D)=\prod_{j=1}^{P}p(Y_{j}|Y_{D_{j}},\Theta_{Y_{j}}) \end{array}$$

where $D_{j}$ is a set of parents for the j th outcome $Y_{j}$ , and $Y_{D_{j}}=\{Y_{i}:i\in D_{j}\}$ , j=1,2,…,P , and $\Theta_{Y_{j}}$ are the parameter space for the local probability distribution of $Y_{j}$ . For a given BN structure D , each local conditional distribution $P(Y_{j}|Y_{D_{j}},\Theta_{Y_{j}})$ is associated with its own parameter space $\Theta_{Y_{j}}$ which can be estimated using either likelihood-based or Bayesian methods. In BNs with categorical nodes, these parameters are represented by conditional probability tables (CPTs). For example, for a categorical node $Y_{j}$ with parents $Y_{D_{j}}$ and a known network structure, the ML estimate (MLE) of each CPT entry corresponds to the relative frequency of each value of $Y_{j}$ given a specific configuration of its parents. In contrast, for BNs with continuous variables, linear Gaussian conditional probability distributions (CPDs), derived from linear regression models, are commonly used for parameter estimation.

### 2.2. MBN

The representation of the BN provided in the previous section works under the assumption of independent observations. However, in the case of hierarchically structured data, such as individuals nested within groups or clusters (multilevel data), or measurement occasions of individuals at different time points (longitudinal data), the assumption of independent observations is violated. As a result, expression (3) is not applicable due to the inclusion of the clustering variable L in multilevel data, or L and time points t in the case of longitudinal data. Previous studies combined BNs with multilevel modeling, which can account for the effects of higher level clustering variables during structural discovery and estimation of probability distribution.^9,10^

This study considers a two-level multilevel dataset, where lower-level observations are nested within higher-level units. In cross-sectional multilevel data, the lower-level units are individuals nested within clusters or groups. In longitudinal data, repeated measurements are nested within individuals. Let $y_{il,j},\;l=1,\ldots ,m;\;i=1,\ldots ,n_{l};\;j=1,\ldots ,p$ denote the i th lower-level observation in the l th higher-level unit for the j th variable or outcome. Specifically, in multilevel data, $y_{il,j}$ represents the i th individual in cluster l , while in longitudinal data, it represents the i th measurement occasion for individual l . The total number of observations is $N=\sum_{l=1}^{m}n_{l}$ , where m is the number of higher-level units (clusters or individuals), and $n_{l}$ is the number of lower-level observations within unit l . In this case, the parent set $D_{j}$ for the outcome $Y_{j}$ is a collection of level-1 (lower-level) and level-2 (higher-level) covariates and the likelihood in expression (3) is updated as follows:

(4) $$\begin{array}{l} p(Y|D)=\prod_{j=1}^{p}p(Y_{j}|Y_{D_{j}},L,\Theta_{Y_{j}}) \end{array}$$

where L is the level-2 clustering variable. According to earlier studies, edges from L to each of the outcome variables were introduced either based on prior knowledge or by using the likelihood ratio test to assess the significance of including the random effects associated with this higher-level clustering variable during structure learning.^9,10^ In the present study, we assume that the higher-level clustering variable has a significant effect and that edges from this variable to the outcome variables are whitelisted. However, this setting does not affect the conditional independence assumptions of BNs. BNs rely on the conditional independence assumption that each variable is independent of its non-descendants $\left(\mathrm{ND}(Y_{j})\right)$ given its parents, that is, $Y_{j}\;⊥\;\mathrm{ND}(Y_{j})\;|\;Y_{D_{j}}$ . In MBN, this assumption is preserved. Each local conditional distribution is estimated using a multilevel modeling framework to account for between-group heterogeneity, such that conditional independence holds given the parent set and the corresponding group-level random effects $Y_{j}\;⊥\;\mathrm{ND}(Y_{j})\;|\;Y_{D_{j}},\;U_{j}$ . The DAG, therefore, continues to encode conditional dependence relationships among variables, while the multilevel structure affects only the parameterization of the local models. Furthermore, this study considers Gaussian and binary outcome variables, respectively. Thus, the local network for the j th Gaussian outcome $y_{il,j}$ $\left(l=1,\ldots ,m;\;i=1,\ldots ,n_{l}\right)$ , representing the measurement of the i th individual in the l th cluster, is given by the following equation:

$$y_{il,j}=\beta_{0,j}+y_{d_{il,j}}^{⊤}\beta_{j}+z_{il,j}^{⊤}u_{l,j}+\epsilon_{il,j}$$

Equivalently, in matrix form,

(5) $$\begin{array}{l} Y_{j}=\beta_{0,j}1+Y_{D_{j}}\beta_{j}+Z_{j}U_{j}+\epsilon_{j}\\ U_{j}∼N\left(0,\Sigma (\theta_{j})\right),\;\epsilon_{j}∼N\left(0,\sigma_{j}^{2}I\right) \end{array}$$

where j indexes the outcome (node) in the MBN, l indexes clusters, and i indexes individuals or repeated measurements within clusters. The matrix $Y_{D_{j}}$ is the fixed-effects design matrix associated with the parent set $D_{j}$ of outcome $Y_{j}$ , and $\beta_{j}$ is the corresponding vector of fixed-effect coefficients. The matrix $Z_{j}$ is the random-effects design matrix, linking observations to the cluster-specific random-effects collected in the vector $U_{j}=(u_{1,j},\ldots ,u_{m,j}{)}^{⊤}$ . The term $\epsilon_{j}$ denotes the individual-level residual errors. The covariance matrix $\Sigma (\theta_{j})$ is symmetric and positive semi-definite, and governs the variability and correlation structure of the cluster-level random effects for outcome j , with $\theta_{j}$ denoting the corresponding variance–covariance parameters. Note that, in the case of longitudinal data, the vector of time points t at which the measurement occasions were taken are included in the design matrices $Y_{D_{j}}$ and $Z_{j}$ .

Furthermore, if $Y_{j}$ is from an exponential family distribution, its mean is linked to a linear predictor through a link function g(.) . For example, if $Y_{j}$ is a binary outcome, the linear predictor $\eta_{j}$ is linked to the outcome variable through a logit link function as follows:

(6) $$p(Y_{j}=1|Y_{D_{j}},L;\Theta_{Y_{j}})=\frac{\exp\;(\eta_{j})}{1+\exp\;(\eta_{j})}$$

where, $\eta_{j}=\beta_{0}+Y_{D_{j}}\beta_{j}+Z_{j}U_{j}$ . In this study, we considered k correlated random effects, resulting in a total of k(k+1)/2 variance–covariance parameters. Thus, the covariance matrix in expression (5) for k correlated random effects for the j th outcome is given by the following equation:

(7) $$\Sigma_{j}=\left(\begin{array}{cccc} \sigma_{0,j}^{2} & \sigma_{01,j} & \cdots & \sigma_{0k,j}\\ \sigma_{01,j} & \sigma_{1,j}^{2} & \cdots & \sigma_{1k,j}\\ \vdots & \vdots & \ldots & \vdots \\ \sigma_{k0,j} & \sigma_{k1,j} & \cdots & \sigma_{k,j}^{2} \end{array}\right)$$

where the diagonal elements are the variances of the random effects, and the off diagonal elements are the covariances between the random effects. The full variance–covariance matrix of the random effects for the p outcomes in the MBN is given by the following equation:

$$\Sigma =\left(\begin{array}{cccc} \Sigma (\theta_{1}) & 0 & \cdots & 0\\ 0 & \Sigma (\theta_{2}) & \cdots & 0\\ \vdots & \vdots & \ddots & \vdots \\ 0 & 0 & \cdots & \Sigma (\theta_{p}) \end{array}\right)$$

In the previous studies, the frequentist approach (MLE method) was used to compute the local distribution in expressions (5) and (6).^9–11^ However, Scutari et al.^11^ have shown that this approach does not perform accurately in small-sample settings, particularly when the number of observations per cluster is limited, even for BNs with only Gaussian outcomes. Motivated by these limitations, we propose a novel approach called INLA-MBN.

### 2.3. INLA-MBN

**Figure 1.**
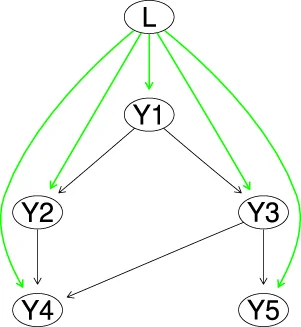
Example of directed acyclic graph (DAG) for a multilevel Bayesian network (MBN).

One of the key characteristics of multilevel data is the presence of within-group dependence, meaning that individuals within the same cluster (e.g. patients in the same hospital) tend to be more similar to each other than to individuals from different clusters. This reflects that observations within the same cluster, sharing a common characteristics, are likely influenced by shared factors or unobserved group-level characteristics. Since we are considering multilevel data in the present study, we assume that an individual from a group carries some information about outcomes observed in other individuals from the same group. When such dependence is evident, we assume that it also extends to the model parameters, for instance, regression coefficients may vary across groups, reflecting heterogeneity in underlying data generating mechanisms. This perspective naturally motivates the use of MBNs, which explicitly accommodate hierarchical data structures while preserving the interpretability of DAGs. Figure 1 illustrates an example DAG corresponding to an MBN.

For this example, the joint probability distribution P(Y|D) factorizes according to the Markov property as follows:

(8) $$\begin{array}{r} P(Y|D)=P(L)P(Y_{1}|L)P(Y_{2}|Y_{1},L)P(Y_{3}|Y_{1},L)\\ P(Y_{4}|Y_{2},Y_{3},L)P(Y_{5}|Y_{3},L) \end{array}$$

**Figure 2.**
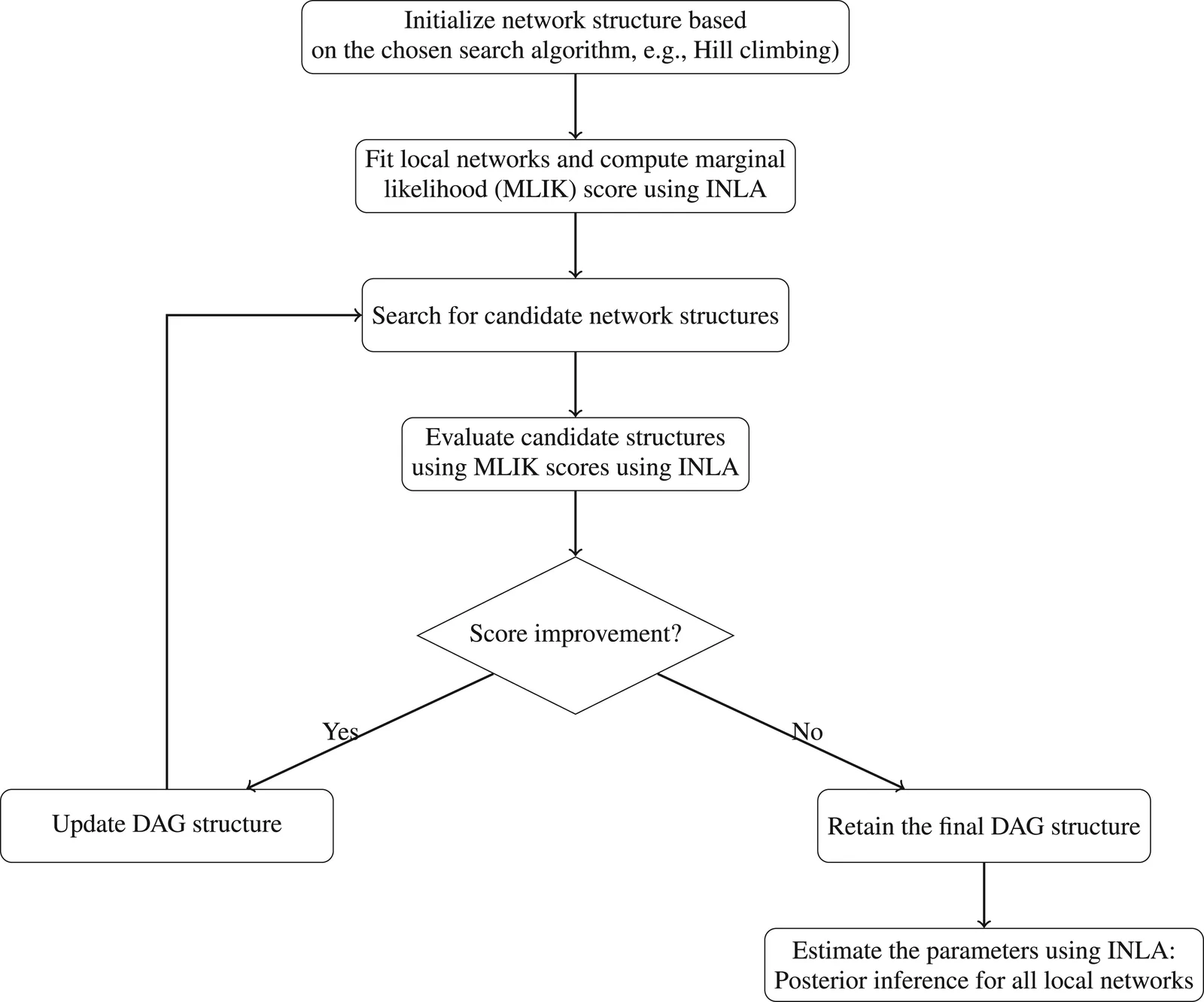
Schematic overview of the multilevel Bayesian networks with the integrated nested Laplace approximation (INLA-MBN) framework.

In previous MBN formulations,^9–11^ the parameters of each local conditional distribution $P(Y_{j}|Y_{D_{j}})$ were estimated using MLE, and structure learning was done using the BIC score. While computationally convenient, this approach becomes unreliable in settings characterized by a small number of clusters and/or small cluster sizes, as the asymptotic assumptions underlying MLE and BIC are violated. In contrast, the hierarchical Bayesian framework enables principled information sharing across groups through hierarchical priors, allowing for partial pooling of parameters. This borrowing of strength stabilizes parameter estimates within each local network and, in turn, can substantially improve the performance of structure inference under limited data conditions. To explicitly model group-level dependence and facilitate such information sharing, we therefore adopt Bayesian hierarchical models for each local conditional distribution in the MBN. However, fully Bayesian inference in complex multilevel models is often computationally prohibitive. To address this challenge, we leverage INLA, which provides fast and accurate approximations to posterior distributions and marginal likelihoods (MLIKs) for latent Gaussian models (LGMs). We, therefore, propose a novel framework called INLA-MBN, which integrates INLA into MBN. In INLA-MBN, each local conditional distribution, $P(Y_{j}|Y_{D_{j}})$ in the network is modeled as a hierarchical Bayesian model and fitted using INLA. Within this framework, INLA is utilized both for parameter estimation and for computing MLIK scores used during structure learning. Accordingly, each local conditional distribution in the MBN is formulated as a LGM, enabling the direct application of INLA for fast and accurate Bayesian inference. This approach combines the structural interpretability of MBNs with the statistical robustness and computational efficiency of Bayesian hierarchical modeling, making it particularly well suited for multilevel data with a small number of clusters and cluster sizes. Figure 2 illustrates the complete workflow of the proposed INLA-MBN framework. The flowchart shows how the global DAG is constructed through iterative score-based structural inference, while each local network corresponding to a node is modeled and fitted independently using INLA.

### 2.4. Local network models as LGMs

In a Bayesian hierarchical model, the prior distributions of certain parameters are specified in relation to other parameters that themselves have prior distributions. This structure allows for multiple levels of uncertainty, with parameters at higher levels influencing the priors of those at lower levels. Thus, when modeling the local networks within the framework of a hierarchical Bayesian model, we specify the generative process by placing prior distributions on all model components, including outcome-level effects and cluster-specific variation. Given the j th outcome $Y_{j}$ , the likelihood depends on two parameter vectors ϕ and θ . Thus, the Bayesian hierarchical model for the local networks that correspond to the j th outcome is given by the following equation:

(9) $$\begin{array}{rl} Y_{j}|\phi_{j},\theta_{j} & ∼p(Y_{j}|\phi_{j},\theta_{j})\\ \phi_{j}|\theta_{j} & ∼p(\phi_{j}|\theta_{j})\\ \theta_{j} & ∼p(\theta ) \end{array}$$

where $p(Y_{j}|\phi_{j},\theta_{j})$ is the likelihood, with the prior distribution $p(\phi ,\theta_{j})$ that is given by the following equation:

(10) $$\begin{array}{r} p(\phi_{j},\theta_{j})=p(\phi_{j}|\theta_{j})p(\theta_{j}) \end{array}$$

where $\theta_{j}$ is the hyperparameter and $p(\theta_{j})$ is the hyper-prior.

In this study, the parameters in the latent field ϕ were assigned Gaussian priors, such that the fixed and random effects in (5) and (6) form a Gaussian latent field. Since the fixed effects are assumed to be independent and the random effects have sparse precision matrices by construction, the latent field is Markovian. The local network models defined in (5) and (6) are LGMs, and we can use INLA for the Bayesian inference of the parameters $\phi_{j}$ and $\theta_{j}$ .^21^

## 3. Structure and parameter inference

Structure learning in BNs aims to identify a DAG that best represents the conditional dependencies among the variables. It consists of two main components: a score function and a search algorithm. The score function evaluates how well a candidate graph fits the observed data. In this study, we use the MLIK score function (see expression (14)), where the score of a full DAG is the sum of the local network scores.

Furthermore, a search algorithm provides a strategy for exploring the space of possible DAG in order to identify the network structure that optimizes the chosen score function. During the learning of the structure of the DAG, the search over candidate network structures is performed using the hill-climbing search algorithm from the bnlearn package in R,^30^ while the corresponding score for each local network is computed by fitting the local models using either MLE or INLA, depending on the chosen score function. The Wishart prior^31^ and the Lewandowski–Kurowicka–Joe (LKJ) prior,^32^ under different hyperparameter settings, were considered during score computation and parameter estimation when the chosen score function was MLIK. Furthermore, conditional parameter estimation of the MBN was done using either MLE or Bayesian inference using INLA depending on the method employed to compute the scores during the structure learning.

### 3.1. INLA

The INLA methodology is a convenient Bayesian computational method that can handle various types of outcomes from the exponential family distributions (e.g. Poisson, Binomial, and Gaussian) through the LGM framework. In the present study, we used the modern INLA version proposed by Van Niekerk et al.^21^ Van Niekerk et al.^21^ showed that the modern INLA framework provides comparable results to MCMC with more than a 100-fold less computational time, especially for complex models such as non-stationary geostatistical models,^33^ joint disease mapping models,^34^ complex survival models,^35^ models for FMRI data,^21,36^ and more.

In the section describing the MBN, $y_{il,j}$ was defined as the j th outcome for the i th individuals in the l th cluster. However, in Bayesian models implemented using INLA, each observation is indexed by $y_{k}$ for k=1,…,N , where k maps to the (i,l) combination of lower and higher level units. Consider the observed data vector that corresponds to an outcome $Y_{j}=(y_{1,j},\ldots ,y_{N,j})$ . Suppose a distribution of $y_{k,j},k=1,\ldots ,N$ for the j th outcome is characterized by its mean $\mu_{k,j}=E(y_{k,j})$ , which is defined as a function of a structured additive predictor $\eta_{k,j}$ through a link function g(⋅) , such that $g(\mu_{k,j})=\eta_{k,j}$ , if $Y_{j}$ is from an exponential family distribution (e.g. see (6)), or $\mu_{k,j}=\eta_{k,j}$ , if $Y_{j}$ is a continuous outcome from a Gaussian distribution (see (5)). The additive linear predictor $\eta_{k,j}$ for the j th outcome is then given by the following equation:

(11) $$\begin{array}{l} \eta_{k,j}=\beta_{0,j}+\sum_{p=1}^{n_{\beta }}\beta_{p,j}Y_{D_{kp,j}}+\sum_{s=1}^{n_{f}}f_{s,j}(z_{ks,j}) \end{array}$$

where $\beta_{0,j}$ is the global mean for the outcome $Y_{j}$ , $Y_{D_{j}}$ is the p th parent of $Y_{j}$ with a linear effect $\beta_{p,j}$ , the $f_{s,j}$ terms are used to model the random effects due to the higher-level clustering variables for the covariate $z_{ks,j}$ , where $z_{j}\in Y_{D_{j}}$ and the latent field $X_{j}$ is then $x_{j}=\{\beta_{0,j},\beta_{j},f_{j}\}$ with a Gaussian prior $x_{j}|\theta_{j}∼N(0,Q_{j}^{-1}(\theta_{j}))$ , where $\theta_{j}$ is the hyperparameter. For a given DAG, each node $Y_{j}$ together with its parent set $Y_{D_{j}}$ defines a local network. In the proposed INLA-MBN framework, each local network is modeled independently using INLA by specifying a LGM with a linear predictor given in (11). Parent variables enter the model either as fixed effects or as group-varying effects, depending on the modeling setting, while group-level dependence induced by higher-level clustering variables is captured through random effects. INLA is then used to perform approximate Bayesian inference for the latent field and associated hyperparameters corresponding to each local network. Thus, the N linear predictor that corresponds to the j th outcome $Y_{j}$ is given by the following equation:

(12) $$\begin{array}{l} \eta_{j}=A_{j}x_{j} \end{array}$$

where A is a sparse design matrix that links the linear predictor to the latent field. Thus, the joint density of the latent field and hyperparameters is given by the following equation:

(13) $$\begin{array}{l} p(x_{j},\theta_{j}|Y_{j})\propto \\ p(\theta_{j})\;p(x_{j}|\theta_{j})\prod_{k=1}^{N}p\left(y_{k,j}|{\left(A_{j}x_{j}\right)}_{k},\theta_{j}\right) \end{array}$$

where $p(\theta_{j})$ is the hyper-prior.

The hyperparameters $\theta_{j}$ govern the precision and correlation structures of the random effects in each local network and are assigned prior distributions to enable hierarchical modeling. These hyperpriors control the degree of shrinkage and allow partial pooling across clusters, thereby facilitating information sharing between groups while preserving within-group variability. Such hierarchical prior specifications are essential for stable inference in settings with a small number of clusters or limited observations per cluster. In this study, INLA was used to calculate the MLIK score of each local network for structure learning and to estimate MBN parameters based on the learned structure. The MLIK score for a local network that corresponds to the node $Y_{j}$ is given by the following equation:

(14) $$\begin{array}{c} p(Y_{j}|M_{j})=\int_{\Theta_{j}}\frac{p(Y_{j},\theta \;\theta_{j},x_{j}|M_{j})}{{\tilde{p}}_{G}(x_{j}|\theta \;\theta_{j},Y_{j},M_{j})}{|}_{x_{j}=x_{j}^{*}(\theta \;\theta_{j}|M_{j})}\;d\theta \;\theta_{j} \end{array}$$

where $M_{j}$ is a Bayesian model corresponding to a local network for the outcome $Y_{j}$ , $\Theta_{j}$ is the parameter space, $x_{j}^{*}(\theta_{j}|M_{j})$ is the posterior mode and ${\tilde{p}}_{G}(x_{j}|\theta_{j},Y_{j},M_{j})$ is a Gaussian approximation to $p_{G}(x_{j}|\theta_{j},Y_{j},M_{j})$ . More detail on the approximation of each term in (13) and (14), and the estimation of the parameters in the latent field and hyperparameters can be found in Van Niekerk et al.^21^

Uncertainty is propagated across the multilevel structure through the hierarchical Bayesian formulation of each local network. Specifically, the ML used for structure learning integrates over both the latent field and hyperparameters, thereby accounting for uncertainty arising from group-level random effects and higher-level variability. As a result, the process of estimating the network structure in INLA-MBN reflects uncertainty at both the individual and cluster levels, rather than relying on point estimates of model parameters. In addition, since correlated random effects were considered in the local networks, both the usual Wishart and LKJ priors were used for comparison. Specifically, the priors on the multivariate random effects’ precision matrix in this study were the Wishart( r , $R^{-1}$ ), where r is the degrees of freedom and R is usually the identity matrix, and the LKJ( η ) prior defined over the correlation matrix, with a shape parameter η , and half-normal or half-Cauchy priors for the marginal precisions.

### 3.2. Performance metrics

The MBN learned using the ML method and INLA for both the inverse-Wishart and LKJ priors was compared to identify the approach that better captures the true network structure and to investigate prior sensitivity for the Bayesian approach. The learned structures were evaluated using the structural Hamming distance (SHD)^37,38^ and F1-score.^39^ Following Scutari et al.,^11^ we used the Kullback–Leibler divergence (KLD) to evaluate the performance of parameter estimation, which assesses how well the model approximated the true joint distribution within each group. The KLD is calculated for each group and summed across all groups as follows:

(15) $$\begin{array}{rl} D_{\mathrm{KL}}^{l}(P||Q) & =\frac{1}{2}\left[\log\;\frac{|\Sigma_{Q}^{l}|}{|\Sigma_{P}^{l}|}-d+\mathrm{tr}\left((\Sigma_{Q}^{l}{)}^{-1}\Sigma_{P}^{l}\right)+(\mu_{Q}^{l}-\mu_{P}^{l}{)}^{T}(\Sigma_{Q}^{l}{)}^{-1}(\mu_{Q}^{l}-\mu_{P}^{l})\right]\\ & \text{then}\\ D_{\mathrm{KL}}(P||Q) & =\sum_{l=1}^{m}D_{KL}^{l}(P||Q) \end{array}$$

where $\mu_{P}^{l}$ and $|\Sigma_{P}^{l}|$ represent the mean and determinant of the covariance matrix for the true MBN for the l th group or cluster, respectively, $\mu_{Q}^{l}$ and $|\Sigma_{Q}^{l}|$ represent the mean and determinant of the covariance matrix for the learned MBN, respectively, and d is the number of nodes that determine the dimension of the multivariate distribution for a given MBN.

The F1-score was utilized for group prediction to evaluate how well the model preserved information about the grouping variable L . For each simulation replicate, posterior class predictions for the grouping variable L were obtained from the fitted MBN. While this performance depends on the learned group-level random effects, it does not directly assess the accuracy of the estimated variance–covariance matrix of random effects. Let the total sample size $n=\sum_{l=1}^{m}n_{l}$ and let the lower level individuals be indexed by i=1,2,…,n , and for each cluster l classification performance was evaluated. True positives $\left({\mathrm{TP}}_{l}\right)$ were defined as the number of individuals correctly assigned to cluster l , false positives $\left({\mathrm{FP}}_{l}\right)$ were defined as the number of individuals incorrectly assigned to the cluster l , and false negative $FN_{l}$ is the number of individuals belonging to cluster l but assigned to a different cluster. Then the cluster specific F1-score was computed as follows:

(16) $$\begin{array}{rl} & {\text{F1}}_{l}=\frac{2*{\mathrm{TP}}_{l}}{2{\mathrm{TP}}_{l}+{\mathrm{FP}}_{l}+{\mathrm{FN}}_{l}}\\ & {\text{F1-Score}}_{L}=\frac{1}{m}\sum_{l=1}^{m}{\text{F1}}_{l} \end{array}$$

The F1-score was also used to evaluate the performance of structure learning in the MBN. In this context, a true positive (TP) corresponds to a directed edge predicted by the MBN that exists in the true network, a false positive (FP) corresponds to a directed edge predicted by the MBN that does not exist in the true network, and a false negative (FN) corresponds to a directed edge that exists in the true network but is not predicted by the MBN. The structure-level F1-score was then computed as F1-Score = 2TP/(2TP+FP+FN). Furthermore, SHD is defined as the number of edge additions, deletions, and orientation changes required to transform one graph into another.

Therefore, the KLD evaluates the fidelity of the parameter estimates to the true joint distribution across groups, while the F1-score assesses the ability of the learned network to retain discriminative information through the grouping variable. The KLD provides insight into the accuracy of parameter estimation across groups; however, in multilevel modeling, the grouping variable should be captured properly for accurate results. In addition, in the case of Bayesian hierarchical models, the priors are not only placed directly on the random effects themselves but also on their associated hyperparameters, specifically the precision, indicating that the KLD by itself might not be appropriate for quantifying the effect of the different priors on the parameter estimates. Thus, using both the KLD and the F1- ${\mathrm{Score}}_{L}$ (classification accuracy) for parameters and group prediction, along with the SHD value and F1-score for structure learning, is important to evaluate the overall performance of the MBN.

## 4. Simulation study

In this section, we conducted a simulation study to compare the performance of MBNs learned using the MLE and INLA. The comparison focuses on both the structure and parameter learning of the MBN. The study considers two-level multilevel data under longitudinal and cross-sectional scenarios. We begin with a longitudinal dataset that includes two correlated random effects and evaluate the methods (MLE and INLA) for Gaussian outcomes, as well as for a mix of Gaussian and binary outcomes. In the next step, we extend this simulation to scenarios with k correlated random effects. The random effects for both longitudinal and cross-sectional multilevel data were simulated considering a symmetrical and positive semidefinite covariance matrix with moderate correlation and reasonable variance between 0.3 and 1.

To assess the robustness of the hill-climbing search procedure, we conducted preliminary experiments with multiple random restarts (20 initializations) in the considered setting (10 nodes). The resulting network structures were identical across restarts, indicating that the optimization was not sensitive to initial values in this setting. Therefore, the main simulation study was conducted without random restarts to reduce computational cost.

### 4.1. Longitudinal data

As part of this simulation study, we considered two longitudinal datasets where measurement occasions are nested within individuals. We aim to explore structure and parameter estimation with partial pooling of information for the Gaussian MBN (only Gaussian nodes) and for the hybrid MBN (Gaussian and binary nodes). In this setting, we used two datasets with 30 and 50 individuals, respectively, where five measurement occasions (taken at i=1,…,5 time points) were nested within each individual. A Wishart prior and an LKJ prior on the precision and correlation matrices were considered, respectively. Thus, we considered multiple simulation conditions to assess the sensitivity of the structure and parameter inference of the MBN under various settings. Specifically, we used two true MBNs with comparable size, sparsity, and overall complexity, while differing in their specific dependency patterns and parent–child configurations. This design allows us to assess whether the performance of the proposed method is robust to variations in the underlying network structure, rather than being driven by a particular configuration. Furthermore, we considered two types of priors (Wishart and LKJ) with both the default R-INLA settings and one user-defined prior for each, and two types of MBNs: a Gaussian MBN and a hybrid MBN. We also generated two datasets for each configuration. This resulted in a total of 32 simulation conditions ( 2×2×2×2×2 ). In addition, we fitted MBNs using the BIC score for comparison, considering eight simulation conditions ( 2datasets×2true MBNs×2MBN types ). As noted by Morris et al.,^40^ commonly used number of iterations in simulation studies evaluating statistical methods range from 500 to 1000. Balancing computational feasibility with the stability of the performance estimates, we generated 500 datasets for each simulation condition.

Synthetic data were generated based on randomly selected MBN DAG structures with 10 nodes, where arcs were included independently for each possible node pair with a probability of 0.2. Each of the local networks was simulated based on the longitudinal mixed-effects model. For both Gaussian and hybrid MBNs, let the j th Gaussian node $y_{il,j}(l=1,2,\ldots ,m;i=1,2,\ldots ,n_{l})$ be the i th measurements of the l th individual, and it was simulated based on a linear mixed-effects model, which is given by the following equation:

$$y_{il,j}=\beta_{0,j}+\beta_{1,j}t_{il,j}+y_{d_{il,j}\beta_{j}}^{⊤}+u_{0l,j}+t_{il,j}u_{1l,j}+\epsilon_{il,j}$$

were, $\beta_{0,j}$ denotes the intercept, $\beta_{1,j}$ represents the average rate of change of $y_{il,j}$ over time, and $t_{il,j}$ is the time associated with the ith measurement of the lth individual. Furthermore, $y_{d_{il,j}}$ denotes a vector of fixed-effect covariates with corresponding regression coefficients $\beta_{j}$ , $u_{0l,j}$ , and $u_{1l,j}$ are random effects associated with random intercept and random slope, respectively. we have considered a correlated random effects between random intercept and random slope with respect to the time points t , therefore

(17) $$U_{j}=\left(\begin{array}{c} u_{0l,j}\\ u_{1l,j} \end{array}\right)∼\mathrm{MVN}\left(\begin{array}{c} \left[\begin{array}{c} 0\\ 0 \end{array}\right],\left[\begin{array}{cc} \sigma_{u_{0,j}}^{2} & \sigma_{01}\\ \sigma_{01} & \sigma_{u_{1,j}}^{2} \end{array}\right] \end{array}\right)$$

Consider the hybrid MBN, the nodes are drawn from Gaussian and Bernoulli distributions, with $n_{l}$ measurement occasions nested within each individual. Thus, for the j th binary outcome, the probability $\mathrm{Pr}(y_{il,j}=1)$ is linked to the linear predictor via a logit link function as follows:

$$\begin{array}{rl} & \eta_{il,j}=\beta_{0,j}+\beta_{1,j}t_{il,j}+y_{d_{il,j}\beta_{j}}^{⊤}+u_{0l,j}+t_{il,j}u_{1l,j}\\ & P_{il,j}=\frac{e^{\eta_{il,j}}}{1+e^{\eta_{il,j}}},\;y_{il,j}∼\mathrm{Bernoulli}(P_{il,j}) \end{array}$$

where $\eta_{il,j}$ and $P_{il,j}$ represent the linear predictor and the conditional probability of success for the j th binary outcome at the i th measurement of the l th individual. A correlated random effects between random intercept and random slope was also considered.

The Wishart and LKJ priors were utilized because the Wishart prior was adopted due to its widespread use and computational convenience, while acknowledging its known sensitivity to hyperparameter choices^41^ and its tendency to jointly regularize variances and correlations. In contrast, the LKJ prior was included as a more flexible alternative that directly targets the correlation matrix and allows separate control over correlation strength. Let Σ is a precision matrix and $\Sigma^{-1}$ represent the variance–covariance matrix in (17). In the case of a Wishart prior the covariance matrix $\Sigma^{-1}$ is parameterized as $\Sigma =LL^{T}$ , where L is a lower triangular matrix which is given by the following equation:

(18) $$L=\left(\begin{array}{cc} \exp\;(\theta_{1}) & 0\\ \theta_{3} & \exp\;(\theta_{2}) \end{array}\right)$$

where the precision matrix Σ follows a Wishart distribution $\Sigma ∼{\text{Wishart}}_{k}(r,R^{-1})$ , where r>k+1 for k random effects. The hyperparameters are $\theta_{1},\theta_{2},\text{and}\;\theta_{3}$ , and the prior parameters are $\left(r,R_{1},R_{2},R_{3}\right)$ (see inla.doc("iidkd") from the R-INLA package for details). We considered two Wishart priors: the informative prior (100, 1, 1, 0), set as the current default option in the R-INLA package, and a user-defined, weakly informative prior (6, 1, 1, 0) (see Table 1). This choice of priors was motivated by the sensitivity of the Wishart prior to the degrees of freedom parameter r .^42^ Larger values of r induce stronger shrinkage of the eigenvalues of the covariance matrix, which may sharply shrink correlations and overly regularize the random-effects structure, especially when data is limited. Conversely, smaller values of r impose weaker regularization, allowing greater flexibility while maintaining identifiability and numerical stability. By including both settings, we explicitly assess the impact of prior informativeness across the two cluster sizes.

**Table 1.** Prior specifications for the simulation study considering longitudinal data.

Label	Prior specification
Informative Wishart	$\Sigma ∼{\text{Wishart}}_{k}(r=100,R^{-1})$ , with R=diag(1,1)
Weakly informative Wishart	$\Sigma ∼{\text{Wishart}}_{k}(r=6,R^{-1})$ , with R=diag(1,1)
Informative LKJ	C∼LKJ(η=13)
Weakly informative LKJ	C∼LKJ(η=10)

LKJ: Lewandowski–Kurowicka–Joe.

To implement this structure, the latent field for a node $Y_{j}$ with a correlated random intercept and random slope is constructed using a stacked indexing strategy. We define an intercept index ranging from 1,2,…,N and slop index randging from N+1,N+2,…,2N . These indices are linked to a shared 2×2 unstructured covariance matrix. This approach ensures that the intercept and slope for each individual are correctly mapped and correlated within the latent field. A code snippet illustrating the construction of these stacked indices and the corresponding f() function calls is provided in the Supplemental Material.

Also, this study considered the LKJ prior on the correlation matrix. Consider the correlation matrix $C=BB^{T}$ , where B is the lower triangular matrix in which the parameterization uses the hypersphere decomposition^43^ (see help(cgeneric_LKJ) from the R-INLA package for details). Then C∼LKJ(η) , where η is the shape parameter. Two LKJ prior settings, η=10 (weakly informative) and η=13 (informative) were used to ensure the robustness of the simulation study (see Table 1).

In this study, we compare structure learning based on the BIC score and the MLIK computed via INLA. While BIC relies on MLE and provides an asymptotic approximation, the MLIK score integrates over the parameter space and incorporates prior information. This comparison is particularly relevant in small-sample settings, where prior assumptions may influence parameter estimation and structure recovery.

**Figure 3.**
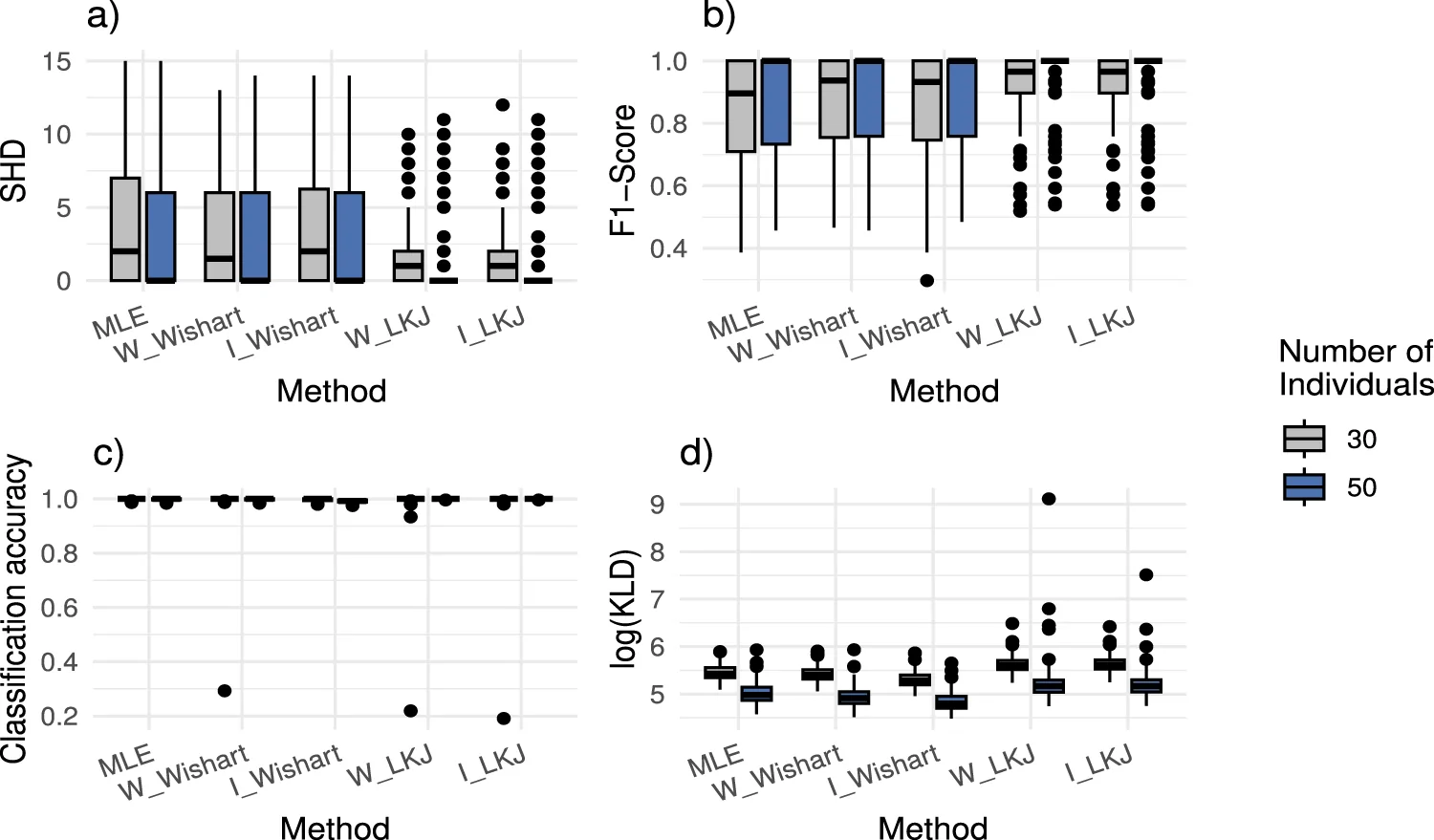
Performance comparison of Gaussian MBN under different estimation methods and prior choices, including MLE, weakly and more informative Wishart priors (W-Wishart and I-Wishart), and weakly and more informative LKJ priors (W-LKJ and I-LKJ), for longitudinal data with varying higher level units. MBN: multilevel Bayesian network; MLE: maximum likelihood estimate; W-LKJ: weakly Lewandowski–Kurowicka–Joe; I-LKJ: informative Lewandowski–Kurowicka–Joe.

We first considered Gaussian MBN and the results of the simulation study indicate that the structure of the MBN learned using INLA with an LKJ prior outperforms the structure based on the Wishart prior and MLE (Figure 3). Notably, in the longitudinal setting with 30 individuals and five repeated measurements per individual, more than 75% of the iterations yielded a SHD below 3 and an F1-score above 0.9 for the MBN with an LKJ prior (Figure 3). Furthermore, the structure based on MLE has a higher SHD and a lower F1-score compared to the structure based on INLA with the Wishart and LKJ priors. Figure 3(c) shows how well the model captures the information about the grouping structure (e.g. individual ID) that is reflected in the data for the observed variables, and all methods captured the information about the grouping variable ID very well, except for a few iterations. Although the LKJ prior yielded superior performance in discovering the true structure, as evidenced by lower SHD and higher F1-scores, it resulted in a slightly higher KLD compared to that of the Wishart prior and MLE. However, as discussed in Section 3.2, the KLD alone does not capture the overall parameter estimation performance. We must also consider the classification accuracy for group prediction. Based on this metric, the LKJ prior preserved information about the grouping variable better than the other methods. In addition, structure learning is a key component of any BN. In this regard, the LKJ priors approximated the true MBN more accurately compared to the other methods.

A similar pattern was observed for the longitudinal setting with 50 individuals and five repeated measurements (Figure 3). Furthermore, the results show that the performance of the structure and parameter estimation based on the LKJ prior increases as the number of individuals in the longitudinal data increases from 30 to 50 (Figure 3). On the other hand, the performance of the MBN structure for the two Wishart priors was comparable for datasets with 30 and 50 individuals (see Figure 3).

For the binary outcome nodes in the hybrid MBN, model identification was achieved by fixing the latent residual variance to $\pi^{2}/3$ , consistent with the standard logistic link function. Because the scale of the fixed effects is conditional on the random effects covariance in nonlinear models, we performed a thorough sensitivity analysis to evaluate the impact of the prior scale on our estimates. We compared parameter recovery across four variance–covariance prior specifications, informative and weakly informative variants of both Wishart and LKJ families. As shown in Tables 2 and 3, the fixed-effect estimates remained robust across these specifications, with absolute bias remaining minimal in most scenarios. While the informative Wishart prior exhibited over-regularization of variance components in small samples (see Tables 2 and 3), the stability of the fixed effects confirm that our substantive conclusions are not artifacts of a specific prior scale. Furthermore, by maintaining an unstructured covariance matrix for each local network models during the structure search, we allowed for maximum flexibility in to account for correlations, ensuring that optimal DAG selection was driven by true improvements in the score rather than restricted scaling.

**Table 2.** Parameter estimates based on the true DAG for MBN with two correlated random effects; a case of longitudinal data with 30 individuals.

			Type of	True					
Child		Parent	estimate	parameter	MLE	I-Wishart	W-Wishart	W-LKJ	I-LKJ
$Y_{1}$	Level I		${\hat{\beta }}_{0}$	0.5	0.42	0.22	0.27	0.32	0.32
		*t*	${\hat{\beta }}_{1}$	−0.3	−0.23	−0.11	−0.16	−0.21	−0.21
		ID	${\hat{\sigma }}_{u_{0}}^{2}$	1.0	4.89	0.03	0.23	0.35	0.35
	Level II	*t*	${\hat{\sigma }}_{u_{1}}^{2}$	0.7	1.09	0.04	0.37	0.62	0.62
			${\hat{\sigma }}_{u_{01}}$	0.42	−1.28	2.7*e*^−3^	0.08	0.11	0.11
$Y_{3}$			${\hat{\beta }}_{0}$	−0.5	−0.21	−0.35	−0.21	−0.12	−0.12
	Level I	*t*	${\hat{\beta }}_{1}$	0.3	0.14	0.15	0.14	0.14	0.14
		$Y_{1}$	${\hat{\beta }}_{2}$	−0.9	−1.39	−1.01	−1.37	−1.79	−1.79
		ID	${\hat{\sigma }}_{u_{0}}^{2}$	0.45	0.38	0.034	0.17	0.23	0.23
	Level II	*t*	${\hat{\sigma }}_{u_{1}}^{2}$	0.35	0.23	0.037	0.23	0.49	0.49
			${\hat{\sigma }}_{u_{01}}$	−0.19	−0.03	1.3*e*^−3^	0.01	0.04	0.04
$Y_{6}$			${\hat{\beta }}_{0}$	1.03	0.93	0.91	0.89	0.92	0.92
		*t*	${\hat{\beta }}_{1}$	−0.27	−0.28	−0.53	−0.30	−0.29	−0.30
	Level I	$Y_{1}$	${\hat{\beta }}_{2}$	0.77	0.82	0.02	0.82	0.79	0.79
		$Y_{5}$	${\hat{\beta }}_{3}$	1.25	1.33	1.52	1.35	1.34	1.34
		ID	${\hat{\sigma }}_{u_{0}}^{2}$	0.70	1.08	0.13	0.83	0.84	0.84
	Level II	*t*	${\hat{\sigma }}_{u_{1}}^{2}$	0.53	0.44	0.22	0.39	0.46	0.46
			${\hat{\sigma }}_{u_{01}}$	0.29	0.18	0.13	0.17	0.18	0.17

DAG: directed acyclic graph; MBN: multilevel Bayesian network; MLE: maximum likelihood estimate; W-LKJ: weakly Lewandowski–Kurowicka–Joe; I-LKJ: informative Lewandowski–Kurowicka–Joe.

**Table 3.** Parameter estimates based on the true DAG for MBN with two correlated random effects; a case of longitudinal data with 50 individuals.

			Type of	True					
Child		Parent	estimate	parameter	MLE	I-Wishart	W-Wishart	W-LKJ	I-LKJ
$Y_{1}$	Level I		${\hat{\beta }}_{0}$	0.5	0.654	0.561	0.647	0.708	0.708
		*t*	${\hat{\beta }}_{1}$	−0.3	−0.144	−0.162	−0.165	−0.184	−0.184
		ID	${\hat{\sigma }}_{u_{0}}^{2}$	1.0	0.322	0.039	0.269	0.313	0.309
	Level II	*t*	${\hat{\sigma }}_{u_{1}}^{2}$	0.7	1.17	0.029	0.826	0.698	0.698
			${\hat{\sigma }}_{u_{01}}$	0.42	0.323	0.014	0.259	0.276	0.266
$Y_{3}$			${\hat{\beta }}_{0}$	−0.5	−0.413	−0.453	−0.404	−0.444	−0.440
	Level I	*t*	${\hat{\beta }}_{1}$	0.3	0.188	0.194	0.192	0.213	0.211
		$Y_{1}$	${\hat{\beta }}_{2}$	−0.9	−0.857	−0.710	−0.865	−0.991	−1.005
		ID	${\hat{\sigma }}_{u_{0}}^{2}$	0.45	0.466	0.033	0.177	0.187	0.166
	Level II	*t*	${\hat{\sigma }}_{u_{1}}^{2}$	0.35	0.711	0.041	0.340	0.584	0.574
			${\hat{\sigma }}_{u_{01}}$	−0.19	−0.576	0.001	−0.107	−0.219	−0.166
$Y_{6}$			${\hat{\beta }}_{0}$	1.03	0.870	0.769	0.859	0.941	0.941
		t	${\hat{\beta }}_{1}$	−0.27	−0.305	−0.374	−0.313	−0.277	−0.276
	Level I	$Y_{1}$	${\hat{\beta }}_{2}$	0.77	0.744	0.694	0.737	0.723	0.723
		$Y_{5}$	${\hat{\beta }}_{3}$	1.25	1.324	1.380	1.330	1.299	1.298
		ID	${\hat{\sigma }}_{u_{0}}^{2}$	0.70	0.826	0.174	0.691	0.690	0.692
	Level II	*t*	${\hat{\sigma }}_{u_{1}}^{2}$	0.53	0.386	0.233	0.366	0.411	0.412
			${\hat{\sigma }}_{u_{01}}$	0.29	0.151	0.163	0.150	0.142	0.139

DAG: directed acyclic graph; MBN: multilevel Bayesian network; MLE: maximum likelihood estimate; W-LKJ: weakly Lewandowski–Kurowicka–Joe; I-LKJ: informative Lewandowski–Kurowicka–Joe.

**Figure 4.**
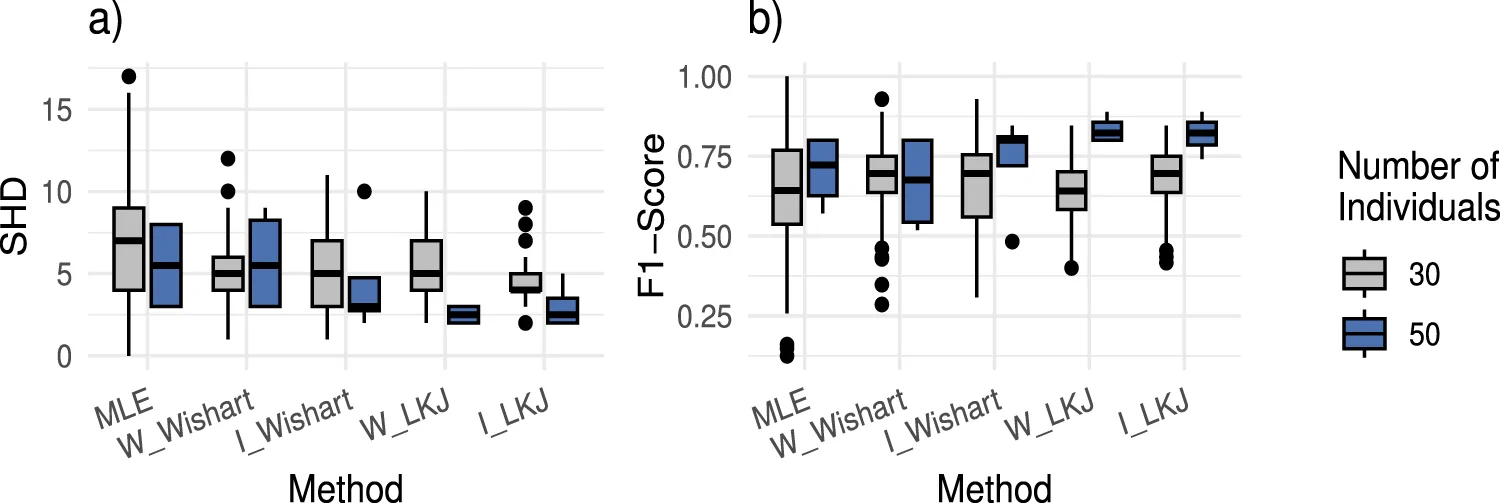
Performance comparison of hybrid MBN under different estimation methods and prior choices, including MLE, weakly and more informative Wishart priors (W-Wishart and I-Wishart), and weakly and more informative LKJ priors (W-LKJ and I-LKJ), for longitudinal data with varying sample sizes. MBN: multilevel Bayesian network; MLE: maximum likelihood estimate; W-LKJ: weakly Lewandowski–Kurowicka–Joe; I-LKJ: informative Lewandowski–Kurowicka–Joe.

For the hybrid MBN, although both the LKJ and Wishart priors exhibit comparable performance for longitudinal data with 30 individuals, the LKJ prior performs slightly better than the Wishart prior. Figure 4(a) shows that the SHD is lower for the hybrid MBN based on the LKJ prior compared to the Wishart prior and MLE. Similarly, the F1-scores for the LKJ priors are higher than those of the MBN with BIC score and MLIK score using Wishart priors, especially for the longitudinal data with 50 individuals (Figure 4(b)). Furthermore, the median F1-score was observed to increase from ∼ 0.7 to ∼ 0.85 as the number of individuals increases from 30 to 50 (Figure 4(b)). Conversely, the performance of structure learning based on the weakly informative Wishart prior decreases when the number of individuals increases from 30 to 50 and the informative Wishart prior performs better than the weakly informative Wishart prior and MLE for data with 50 individuals (Figure 4). A similar result was observed for true MBN 2 in both Gaussian and hybrid MBNs for longitudinal data with 30 and 50 individuals (see Supplemental Figures S1 and S2).

The parameter estimates for some randomly selected local networks for a randomly selected hybrid MBN for longitudinal data with 30 individuals are presented in Table 2. The informative Wishart prior tends to underestimate elements of the variance–covariance matrix, especially for the binary outcomes. In contrast, the MLE approach tends to overestimate some elements of the variance–covariance matrix. Furthermore, although the LKJ prior better approximates the true MBN, the performance of the method for hybrid MBNs indicates that the hybrid MBN is a more complex problem than the Gaussian MBN, as expected.

Table 3 presents the parameter estimates for some randomly selected local networks for the hybrid MBN with 50 individuals. Although the parameters estimated using the LKJ prior were closer to the true values, the Wishart prior and MLE also produced comparable results, except for the random effects under the informative Wishart prior. The accuracy of the random-effect estimates with the informative Wishart prior improved as the number of individuals increased from 30 to 50 (see Tables 2 and 3).

### 4.2. Multilevel cross-sectional data

This section focuses on a multilevel cross-sectional dataset in which individuals are nested within clusters, with five individuals per cluster. We investigate structure and parameter learning in MBNs with k correlated random effects, considering both Gaussian and hybrid MBNs. Similar to the longitudinal simulation study, the analysis of multilevel cross-sectional data employs both frequentist and Bayesian approaches. For both Gaussian and hybrid MBNs, k correlated random effects were specified within clusters comprising 30 and 50 levels, each containing five individuals. The network consists of 10 nodes, with arcs included independently with a probability of 0.2. The j th Gaussian node $y_{il,j}(l=1,\ldots ,m;i=1,2,\ldots ,n_{l})$ was simulated in the linear mixed-effects model framework, which is given by the following equation:

$$y_{il,j}=\beta_{0,j}+y_{d_{il,j}}^{⊤}\beta_{j}+z_{il,j}^{⊤}u_{l,j}+\epsilon_{il,j}$$

and the j th binary outcome was simulated using

$$\begin{array}{rl} \eta_{il,j} & =\beta_{0,j}+y_{d_{il,j}}^{⊤}\beta_{j}+z_{il,j}^{⊤}u_{l,j}\\ P_{il,j} & =\frac{e^{\eta_{il,j}}}{1+e^{\eta_{il,j}}},\;y_{il,j}∼\mathrm{Bernoulli}(P_{il,j}) \end{array}$$

where

(19) $$\left(\begin{array}{c} u_{0l,j}\\ u_{1l,j}\\ \vdots \\ u_{kl,j} \end{array}\right)∼\mathrm{MVN}\left(\begin{array}{c} \left[\begin{array}{c} 0\\ 0\\ \vdots \\ 0 \end{array}\right],\left[\begin{array}{cccc} \sigma_{0,j}^{2} & \sigma_{01,j} & \ldots \sigma_{0k,j}\\ \sigma_{01,j} & \sigma_{1,j}^{2} & \ldots \sigma_{1k,j}\\ \vdots & \vdots & \vdots & \\ \sigma_{0k,j} & \sigma_{1k,j} & \ldots \sigma_{k,j}^{2} \end{array}\right] \end{array}\right)$$

and $y_{d_{il,j}}$ is the fixed-effects design vector, $\beta_{j}$ is a vector of fixed-effect coefficient, $z_{il,j}$ are the design vectors associated with the k correlated random effects $u_{l,j}$ .

As it was done for the longitudinal data simulation study, we also examined the sensitivity of the structure and parameter learning of the MBN under different prior settings on the precision and correlation matrix, using the Wishart and LKJ priors, respectively, as presented in Table 1. In the frequentist approach, we applied MLE to compare the structural discovery and parameter estimation performance of the two methods (MLE and INLA) for a small sample setting.

Two prior settings for both Wishart and LKJ priors were considered (see Table 4). In the case of the Wishart prior, the informative prior $\left(r=100,\underset{k\text{ times}}{\underbrace{1,1,\ldots ,1}},0,0,\ldots \right)$ , and a weakly informative prior $\left(r=6,\underset{k\text{ times}}{\underbrace{1,1,\ldots ,1}},0,0,\ldots \right)$ were used for local networks with >=3 correlated random effects, and $\left(r=10,\underset{k\text{ times}}{\underbrace{1,1,\ldots ,1}},0,0,\ldots \right)$ were used for local networks with <3 correlated random effects. For the LKJ priors, we considered the weakly informative prior where η=10 , and a more informative prior with η=12 . The practical implementation follows the same indexing logic as in the longitudinal data framework, extending the latent field to a size of kN . A code snippet illustrating the construction of the indices for *k* correlated random effects that correspond to the f() function is provided in the Supplemental Material.

**Table 4.** Prior specifications for the simulation study considering multilevel cross-sectional data.

Label	Prior specification
Informative Wishart	$\Sigma ∼{\text{Wishart}}_{k}(r=100,R^{-1})$ , with R=diag(1,1,…,1)
Weekly informative Wishart	$\Sigma ∼{\text{Wishart}}_{k}(r=6,R^{-1})$ , with R=diag(1,1,…,1) , for ≥3 random effects
	$\Sigma ∼{\text{Wishart}}_{k}(r=10,R^{-1})$ , with R=diag(1,1,…,1) , for <3 random effects
Informative LKJ	C∼LKJ(η=12)
Weekly informative LKJ	C∼LKJ(η=10)

LKJ: Lewandowski–Kurowicka–Joe.

Furthermore, we considered two different true MBN structures to check the sensitivity of structural discovery and the parameter estimation of the MBN. The variance and covariance parameters for the covariance matrix were set from 0.3 to 1, with moderate correlation. Thus, similar to the simulation study for the longitudinal data, the total number of simulation conditions considered for the multilevel cross-sectional data was 40 for the Gaussian and hybrid MBNs.

**Figure 5.**
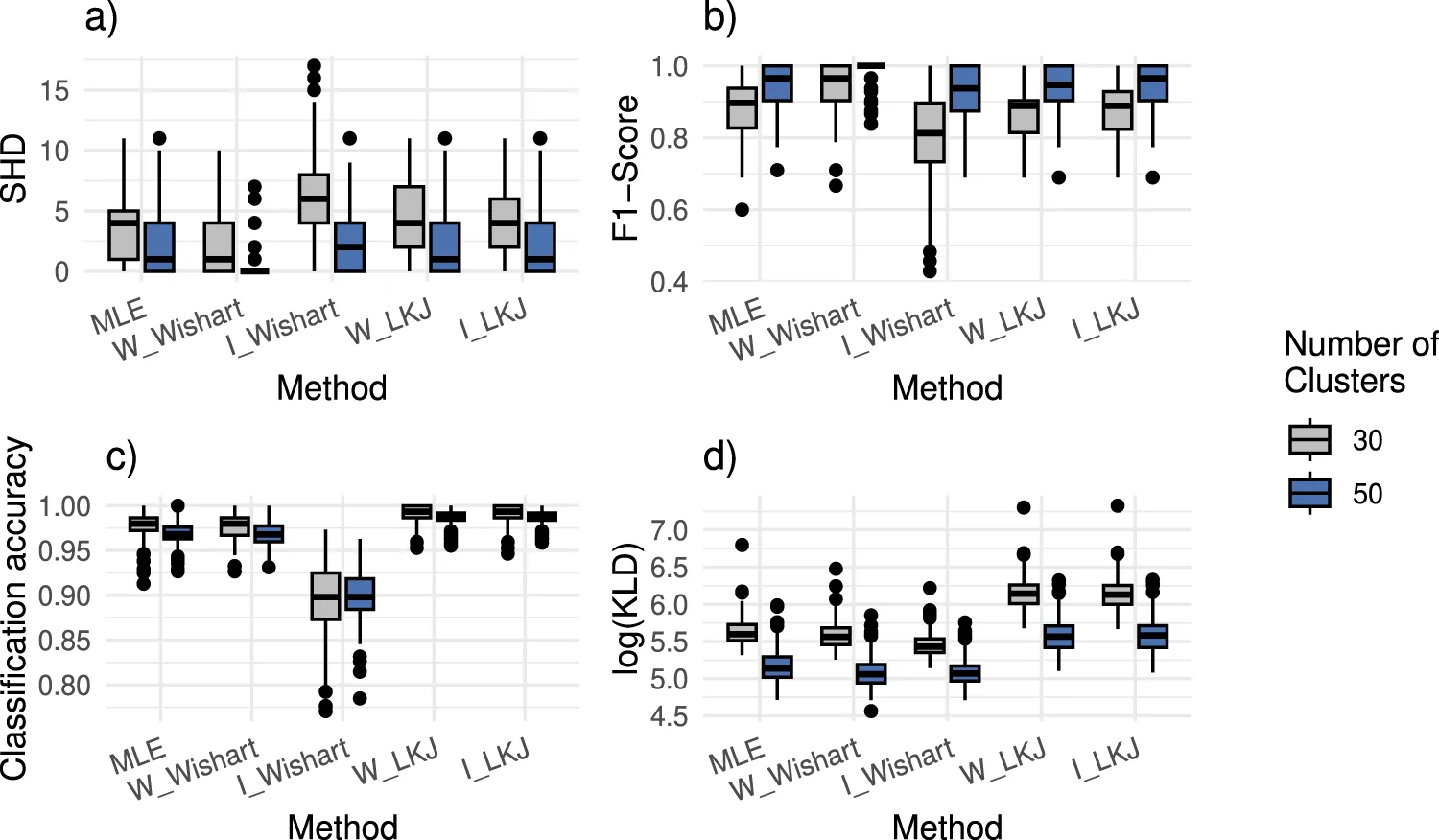
Performance comparison of Gaussian MBN under different estimation methods and prior choices, including MLE, weakly and more informative Wishart priors (W-Wishart and I-Wishart), and weakly and more informative LKJ priors (W-LKJ and I-LKJ), for cross-sectional multilevel data with varying higher level units. MBN: multilevel Bayesian network; MLE: maximum likelihood estimate; LKJ: Lewandowski–Kurowicka–Joe; W-LKJ: weakly Lewandowski–Kurowicka–Joe; I-LKJ: informative Lewandowski–Kurowicka–Joe.

Structure learning is a fundamental step in BN modeling. A poorly learned structure can result in misleading conclusions by creating spurious dependencies or missing critical ones. This can lead to illogical interpretations by suggesting a connection between completely unrelated phenomena. In our simulation, the weakly informative Wishart prior consistently produced highly accurate structures in the case of k correlated random effects, compared to the structure learned from the informative Wishart prior, LKJ priors, and MLE (Figure 5). Specifically, the structure is almost perfectly learned for more than 50% of the iterations for multilevel data with 30 clusters (Figure 5(a) and (b)). In addition, the true DAG structure is retained for almost all iterations for multilevel data with 50 clusters (Figure 5(a) and (b)).

The classification accuracy for the grouping variable exceeds 0.95 except for the informative Wishart prior (Figure 5(c)). This metric shows the ability of MBN to recover the true grouping structure. Although it does not directly assess the accuracy of the estimated variance–covariance matrix of random effects, it depends on the learned group-level random effects. Values close to zero indicate that the model has limited ability to recover the hierarchical structure of the data, which may lead to poor estimation of group-level random effects across local networks. Conversely, probabilities close to 1 indicate that, the information about the grouping structure is well captured for each local network. The result shows that, MBN is sensitive to this parameter, especially for MBN with a number of correlated random effects in a small sample scenario. For example, for multilevel cross-sectional data with *k* correlated random effects, the probabilities for the classification accuracy decrease to around 0.8 for data with 30 clusters under more informative Wishart prior (Figure 5(c)). This occurs because more informative Wishart prior underestimates the elements of the variance–covariance matrix when there are more than two correlated random effects and limited information in the dataset. The effect is reflected in the structure learning performance, as measured by SHD and F1-score (Figure 5(a) and (b)). For multilevel data with 30 clusters, more informative Wishart prior performs poorly compared to MLE and other priors (Figure 5(a) and (b)). In BNs, accurate structural discover is critical, as misleading structures can lead to incorrect parameter estimates and potentially wrong scientific conclusions. Therefore, this study highlights the importance of carefully choosing the prior on the random effects of each local network, especially in small sample scenarios.

Moreover, the parameters of the MBN are better learned using the Wishart priors. Thus, based on SHD, F1-score for the structure, classification accuracy for group prediction, and KLD, the weakly informative Wishart prior best approximates the true network (Figure 5). These results suggest that the weakly informative Wishart prior is well suited for structure learning under this simulation setting. Moreover, it was observed that the accuracy of the structural discovery increased when the number of groups increase across all estimation method and prior settings (Figure 5). The parameters estimated using the Wishart prior are closer to the true parameters compared to the parameters estimated using the MLE and LKJ priors (Figure 5(d)).

**Figure 6.**
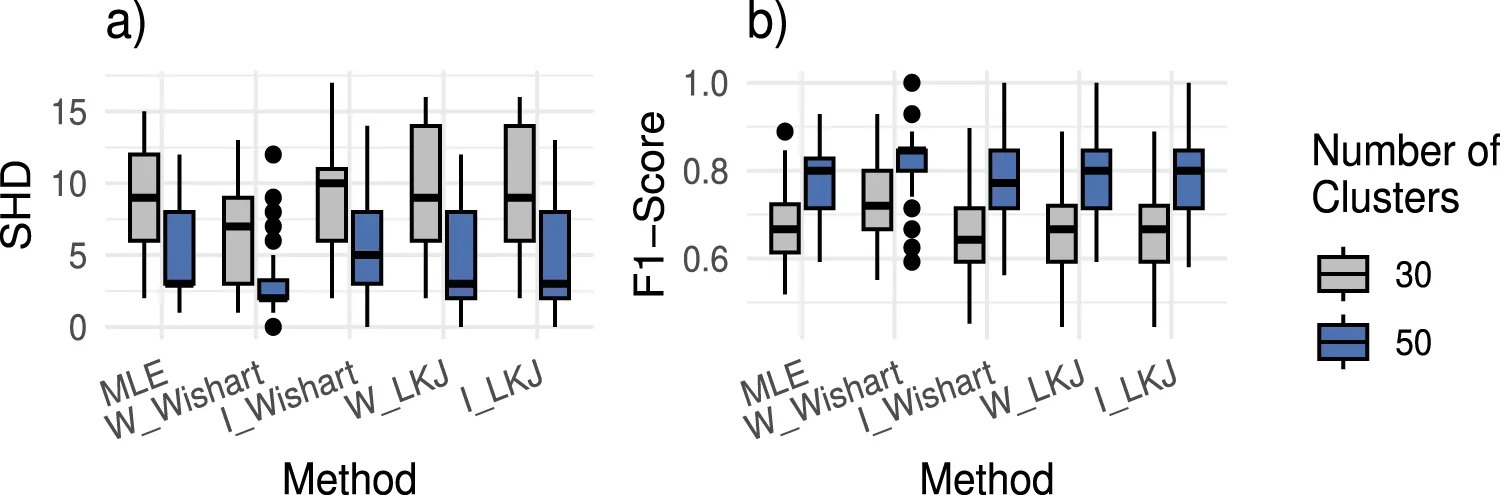
Performance comparison of hybrid MBN under different estimation methods and prior choices, including MLE, weakly and more informative Wishart priors (W-Wishart and I-Wishart), and weakly and more informative LKJ priors (W-LKJ and I-LKJ), for cross-sectional multilevel data with varying higher level units. MBN: multilevel Bayesian network; MLE: maximum likelihood estimate; LKJ: Lewandowski–Kurowicka–Joe; W-LKJ: weakly Lewandowski–Kurowicka–Joe; I-LKJ: informative Lewandowski–Kurowicka–Joe.

The result for hybrid MBN with k correlated random effects indicates that the structure learned using INLA with the less informative Wishart prior exhibits a lower SHD and a higher F1-score compared to the informative Wishart prior, the LKJ priors and the MLE (see Figure 6). Furthermore, it is observed that the F1-score for the MBN based on the weakly informative Wishart prior is >0.7 and 0.85 for more than 50% of the iterations for data with 30 and 50 clusters, respectively. However, similar to the longitudinal data scenario, the performance of hybrid MBNs with k correlated random effects is lower than that of Gaussian MBNs with k correlated random effects. The findings indicate a significant improvement in the performance of retaining the true DAG as the number of clusters increases from 30 to 50. For instance, the median F1-score for the MBN with weakly informative Wishart prior increases from 0.65 for 30 groups to 0.80 for multilevel data comprising 50 groups (Figure 6(b)).

The results of the simulation study for true MBN 2, for both Gaussian and hybrid MBNs, show a similar performance pattern across all simulation scenarios as observed for true MBN 1. This indicates that the performance of the MBN is consistent across different network structures with comparable sparsity levels (see Figures S3 and S4 in the Supplemental Material). In addition, the accuracy of the parameter estimates improves as the number of groups increases (see Tables S1 and S2 in the Supplemental Material).

## 5. Application to real data

This section illustrates the structure and parameter inference of MBN using secondary data from children with severe acute malnutrition (SAM) who were followed up at randomly selected primary clinics in Addis Ababa, Ethiopia. The data originate from a real-world clinical dataset collected in 2023 at four clinics (Bole, Gergi, Kazanchis, and Kirkos). A total of 120 children with at least five follow-up measurements were included in the analysis. Ethical approval for this study was obtained from the institutional review board of Addis Ababa health bureau.

Exploratory data analysis was conducted prior to model fitting to assess the suitability of the proposed model for the data. The results indicate that the proportions of diarrhea, dehydration, acute gastroenteritis (AGE), and pneumonia vary across children (see Figure S5 in the Supplemental Material). Furthermore, individual and mean profile plots of WHZ over time highlight notable variation between subjects (see Figure S6 in the Supplemental Material). These suggest that the MBN framework is appropriate for modeling conditional dependencies among multiple outcomes while accounting for subject-level variation. In addition, summary statistics for all variables considered in this study are provided in Table S3 in the Supplemental Material.

According to WHO guidelines, these children received ready-to-use therapeutic food (RUTF) based on their weight, and the children were followed at an interval of one week (an average of 7 days). The data include a continuous outcome, weight for height (WHZ), and binary outcomes: diarrhea, AGE, pneumonia, and dehydration. Each of these variables was categorized as ‘‘0” if the child did not have the disease and ‘‘1” if the child had the disease. We included the child’s age in months (ranging from 6 to 59) and gender, but all incoming edges to age and gender were blacklisted to enhance the robustness of the structure learning and maintain theoretical validity.

The dataset used in this section did not contain missing values. Nevertheless, it is worth noting that the proposed MBN framework naturally accommodates missing data. In a multilevel modeling context, missing values in either the response or predictors can be handled through the hierarchical model structure, which allows for joint estimation of parameters using all available data. Furthermore, in a fully Bayesian setting, missing values can be treated as additional unknown parameters and estimated jointly with the model parameters, propagating uncertainty due to missingness throughout the network. This approach is analogous to multiple imputation but implemented within the model, ensuring that dependencies across repeated measurements or visits are appropriately accounted for. Extensions to the INLA framework have been proposed for both continuous and categorical covariates, allowing missing values to be modeled jointly rather than independently.^44^ Therefore, even if missing data were present, our framework could handle them in a model-based manner without requiring ad-hoc imputation.

Children with SAM are expected to be vulnerable to various comorbidities, and RUTF was provided to treat SAM. Therefore, the interaction between time and RUTF (time*RUTF) was considered during the structure and parameter estimation. The edges from RUTF to all other variables were whitelisted to reflect the assumption that RUTF can potentially influence other outcomes through WHZ improvement, but not the other way around. In addition, all incoming edges from other outcomes to RUTF were blacklisted to ensure that the model does not erroneously suggest that these outcomes predict whether children should receive RUTF or not, since RUTF was provided to all children to prevent SAM. All incoming edges to gender and age were also blacklisted, as these variables are inherent demographic characteristics and cannot be influenced by other factors. Furthermore, a random restart procedure with 20 initializations was used during structure learning to improve accuracy.

**Figure 7.**
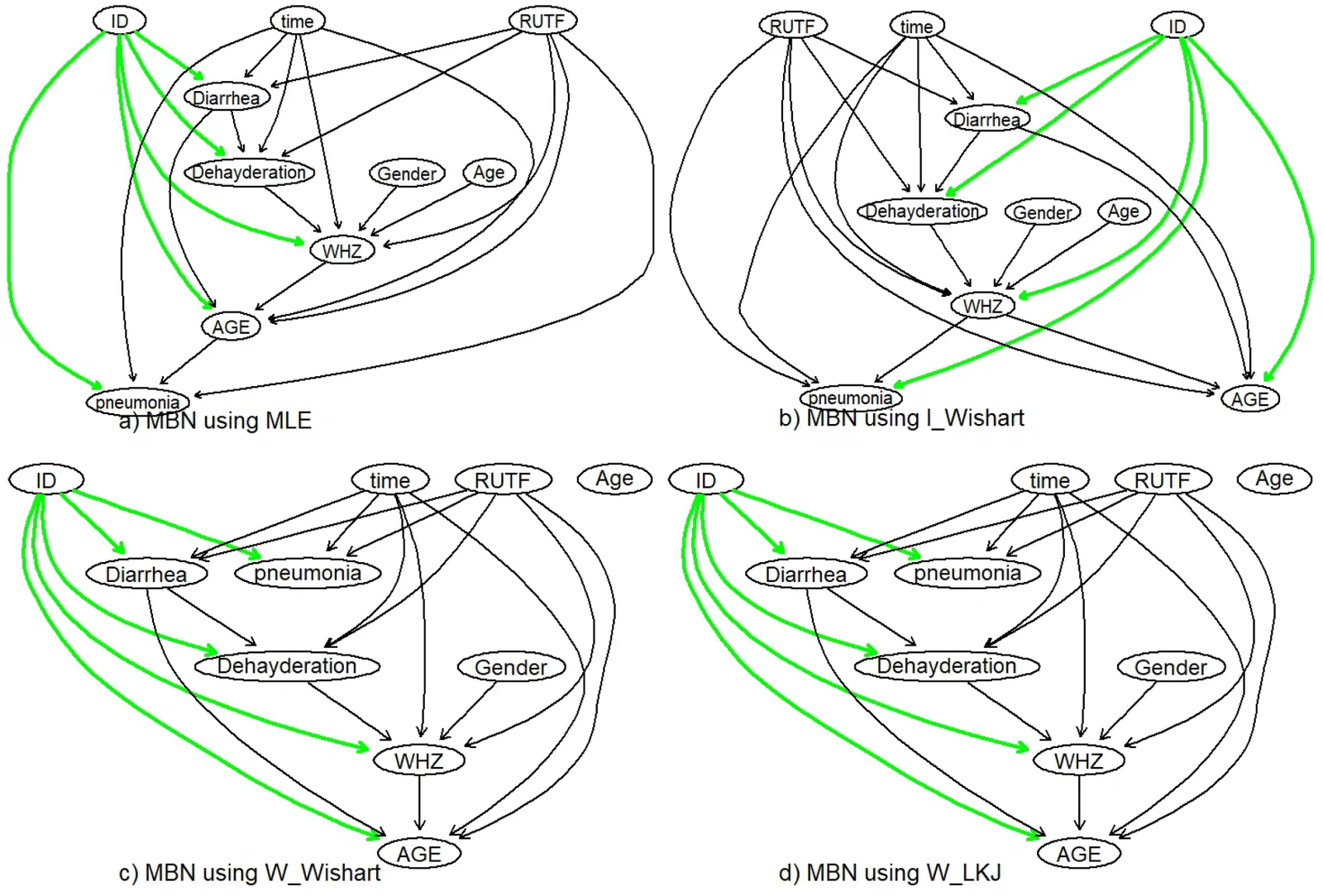
Multilevel Bayesian network for the real child morbidity data using Bayesian inference and MLE. Wishart-S (informative Wishart prior), Wishart-W (weakly informative Wishart prior), and LKJ-W (weakly informative LKJ prior). MLE: maximum likelihood estimate; LKJ: Lewandowski–Kurowicka–Joe.

The results of the structure and parameter learning of MBN based on the proposed method are given in Figure 7 and Table 5, respectively. We considered MLE, INLA with the two Wishart priors, and INLA with the two LKJ priors (Table 1) to calculate the score of local networks during structure learning and to compute the parameters based on the learned structure. According to the results of the simulation study, both the LKJ priors performed similarly when the number of individuals increased to 50. Thus, since the number of individuals for this data is above 50, we only considered the weekly informative LKJ prior to learn the structure and parameters of MBN for the real dataset.

**Table 5.** Parameter estimates of the MBN for the real child morbidity data using MLE and Bayesian inference, corresponding to the learned DAG structure shown in Figure 7.

			Type of	Estimate			
Child		Parent	Estimate	I-Wishart (95% CrI)	W-Wishart (95% CrI)	W-LKJ (95 % CrI)	MLE (95% CI)
			${\hat{\beta }}_{0}$	−1.414 (−1.783, −1.044)	−2.008 (−2.570, −1.445)	−1.309 (−1.672, −0.803)	−2.533 (−3.374, −1.693)
	Level I	Time	${\hat{\beta }}_{1}$	−0.060 (−0.148, 0.029)	−0.136 (−0.264, −0.008)	−0.544 (−0.817, −0.272)	0.103 (−0.050, 0.257)
Diarrhea		RUTF	${\hat{\beta }}_{2}$	0.023 (0.006, 0.041)	0.034 (0.013, 0.056)	0.042(0.020, 0.064)	0.021 (−0.005, 0.046)
		Time * RUTF	${\hat{\beta }}_{3}$	−0.006 (−0.010, −0.002)	−0.007 (−0.012, −0.002)	−0.007 (−0.014, −0.001)	−0.010(−0.0168, −0.003)
	Level II	ID	${\hat{\sigma }}_{u_{0}}^{2}$	0.010	3.459	0.060	5.573
		Time	${\hat{\sigma }}_{u_{1}}^{2}$	0.011	0.061	0.505	0.019
			${\hat{\sigma }}_{u_{01}}$	0.0005	−0.084	0.124	−0.209
			${\hat{\beta }}_{0}$	−2.324 (−2.816, −1.832)	−5.099(−4.227, −2.962)	−2.68 (−3.242, −2.117)	−3.322 (−4.497, −2.467)
		Time	${\hat{\beta }}_{1}$	−0.132 (−0.247, −0.017)	0.361 (−0.001, 0.334)	−1.007(−1.326, −0.688)	−0.061 (−0.230, 0.129)
	Level I	RUTF	${\hat{\beta }}_{2}$	−0.008 (−0.030, 0.014)	0.112 (0.022, 0.073)	0.013 (−0.012, 0.038)	−0.028 (−0.061, 0.001)
Dehydration		Diarrhea: Yes	${\hat{\beta }}_{3}$	2.677 (2.182, 3.172)	4.576 (3.522, 4.819)	3.621 (2.957, 3.621)	3.295 (2.522, 4.205)
		Time * RUTF	${\hat{\beta }}_{3}$	−0.001 (−0.006, −0.001)	−0.017 (−0.019, −0.006)	0.000 (−0.009, 0.009)	0.000 (−0.010, 0.004)
	Level II	ID	${\hat{\sigma }}_{u_{0}}^{2}$	0.010	2.548	0.017	5.222
		Time	${\hat{\sigma }}_{u_{1}}^{2}$	0.010	0.074	0.007	0.031
			${\hat{\sigma }}_{u_{01}}$	0.0001	−0.184	0.002	−0.03
			${\hat{\beta }}_{0}$	−1.658 (−1.982, −1.334)	−2.318 (−2.644, −1.993)	−2.311 (−2.595,−2.026)	−1.636 (−2.080, −1.189)
		Time	${\hat{\beta }}_{1}$	0.180 (0.149, 0.211)	0.182 (0.143, 0.221)	0.184 (0.003, 0.364)	0.160 (0.121, 0.200)
		RUTF	${\hat{\beta }}_{2}$	0.027 (0.022, 0.032)	0.026 ( 0.021, 0.031)	0.025 (0.020, 0.030)	0.028 (0.024, 0.033)
		Dehydration: Yes	${\hat{\beta }}_{5}$	−0.435 (−0.599, −0.272)	−0.473 (−2.644, −1.993)	−0.470 (−0.634, −0.305)	−0.422 (−0.590, −0.255)
	Level I	Age	${\hat{\beta }}_{3}$	−0.029 (−0.039, −0.019)	–	–	−0.031 (−0.045, −0.017)
WHZ		Gender: Male	${\hat{\beta }}_{4}$	−0.788 (−1.083, −0.492)	−0.797 (−1.225, −0.367)	−0.815 (−1.198, −0.432)	−0.789 (−1.191, −0.376)
		Time * RUTF	${\hat{\beta }}_{6}$	−0.001 (−0.002, 0.000)	−0.001 (−0.002, 0.000)	−0.001 (−0.002, 0.000)	−0.0004 (−0.002, 0.000)
	Level II	ID	${\hat{\sigma }}_{u_{0}}^{2}$	0.610	1.456	0.097	1.374
		Time	${\hat{\sigma }}_{u_{1}}^{2}$	0.012	0.028	0.002	0.016
			${\hat{\sigma }}_{u_{01}}$	−0.017	−0.054	−0.003	−0.048
			${\hat{\beta }}_{0}$	−4.219 (−5.306, −3.131)	−14.948 (−7.344, −3.518)	0.049 (−0.138, 0.236)	−6.250 (−8.365, −4.135)
		Time	${\hat{\beta }}_{1}$	−0.003 (−0.196, 0.191)	−1.674 (−0.451, 0.354)	−0.012 (−0.191, 0.167)	0.334 (0.020, 0.648)
		RUTF	${\hat{\beta }}_{2}$	−0.022 (−0.061, 0.017)	−0.022 (−0.076, 0.033)	−0.003 (−0.004, −0.001)	−0.061 (−0.131, 0.009)
AGE	Level I	WHZ	${\hat{\beta }}_{3}$	−0.547 (−0.806, −0.287)	−0.765 (−1.241, −0.289)	−0.028 (−0.045, −0.011)	−0.631 (−1.131, −0.130)
		Diarrhea: Yes	${\hat{\beta }}_{4}$	2.506 (1.817, 3.194)	3.045 (1.964, 4.127)	0.135 (0.096, 0.174)	3.827 (2.498, 5.156)
		Time * RUTF	${\hat{\beta }}_{4}$	−0.011 (−0.023, 0.002)	0.015 (−0.037, 0.010)	0.000 (0.000, 0.001)	−0.023 (−0.045, 0.000)
		ID	${\hat{\sigma }}_{u_{0}}^{2}$	0.010	5.539	0.003	8.385
	Level II	Time	${\hat{\sigma }}_{u_{1}}^{2}$	0.010	0.135	5.8*e*^−5^	0.016
			${\hat{\sigma }}_{u_{01}}$	6.3*e*^−6^	−0.227	−3.4*e*^−5^	−0.373
			${\hat{\beta }}_{0}$	−3.085 (−3.880, −2.290)	−1.823 (−2.479, −1.165)	0.185 (0.004, 0.367)	−2.417 (−3.365, −1.469)
		Time	${\hat{\beta }}_{1}$	−0.106 (−0.298, 0.086)	−0.535 (−1.078, 0.008)	−0.023 (−0.202, 0.156)	−0.492 (−1.437, 0.452)
		RUTF	${\hat{\beta }}_{2}$	0.019 (−0.004, 0.042)	0.023 (−0.011, 0.057)	−0.002 (−0.004, −0.001)	0.022 (−0.028, 0.073)
Pneumonia		WHZ	${\hat{\beta }}_{3}$	−0.460 (−0.666, −0.254)	–	–	–
		AGE: Yes	${\hat{\beta }}_{3}$	–	–	–	−0.982 (−2.694, 0.730)
		Time * RUTF	${\hat{\beta }}_{3}$	−0.006 (−0.016, 0.003)	−0.014 (−0.041, 0.013)	0.000 (0.000, 0.001)	−0.024 (−0.077, 0.028)
		ID	${\hat{\sigma }}_{u_{0}}^{2}$	9.9*e*^−3^	4.575	0.005	10.126
	Level II	Time	${\hat{\sigma }}_{u_{1}}^{2}$	0.011	0.29	8.2*e*^−5^	0.478
			${\hat{\sigma }}_{u_{01}}$	1.3*e*^−4^	0.177	−5.9*e*^−4^	−1.839

*CrI: credible interval; CI: confidence interval; MBN: multilevel Bayesian network; MLE: maximum likelihood estimate; DAG: directed acyclic graph; W-LKJ: weakly Lewandowski–Kurowicka–Joe; RUTF: ready-to-use therapeutic food; WHZ: weight for height; AGE: acute gastroenteritis.

From a clinical perspective, the structure learned based on the MLIK score using INLA with the informative Wishart prior is more plausible, as it reflects the well-established understanding that WHZ directly compromises immune function and increases children’s susceptibility to both pneumonia and AGE (Figure 7(b)). Furthermore, the relationship between diarrhea and AGE is reversed across all methods (Figure 7). In addition, it is observed that the weakly informative Wishart and LKJ priors missed the edges Age⟶WHZ and WHZ⟶pneumonia (Figure 7(c) and (d)). On the other hand, a serial relationship WHZ→AGE→Pneumonia was observed in the structure learned using MLE (Figure 7(a)). This structure implies the conditional independence WHZ⊥Pneumonia∣AGE , meaning that WHZ and pneumonia are independent given AGE. While this type of relationship may occur in rare cases, it is less consistent with the clinically plausible direct relationship between WHZ and pneumonia, as revealed by the structure based on the informative Wishart prior.

Table 5 presents parameter estimates from MLE and Bayesian inference (Wishart and LKJ priors), corresponding to the learned DAG structure shown in Figure 7. To aid interpretation, we focus on selected nodes in the learned DAG (Figure 7(b)). For example, WHZ depends on dehydration, gender, and age, in addition to the whitelisted effects of RUTF and time. The corresponding parameter estimates in Table 5, based on the informative Wishart prior, quantify the strength and direction of these conditional dependencies. For instance, it was observed that WHZ increased over time ( ${\hat{\beta }}_{1}=0.180$ ), reflecting overall nutritional recovery. RUTF intake showed a positive association with WHZ at baseline ( ${\hat{\beta }}_{2}=0.027$ ). However, the negative interaction between time and RUTF ( ${\hat{\beta }}_{6}=-0.001$ ) indicates that the rate of WHZ improvement over time reduces slightly as RUTF intake rises. This negative time by RUTF interaction may partly reflect irregular follow-up patterns for most of the children under follow-up. While WHO guidelines recommend weekly visits, we observed considerable variation in the timing of visits, and some children had extended intervals between measurements. Holding other variables constant, children who were dehydrated had, on average, a 0.435 unit lower WHZ compared with children who were not dehydrated. The remaining nodes can be interpreted similarly.

## 6. Discussion and conclusion

This research investigated the structure and parameter learning of MBN utilizing MLE and INLA under two distinct types of priors and settings, particularly in a small group size multilevel data scenario. This is the first study to implement BNs for multilevel data using the efficient Bayesian framework of INLA. Although a previous study utilized INLA within the context of BNs, its focus was limited to two-level multilevel data considering a single random effect.^45^ In contrast, the present study expands the application of INLA-based MBNs in two significant aspects. Firstly, it addresses longitudinal data incorporating two correlated random effects, facilitating the modeling of within-subject dependencies over time and variability across different subjects. Secondly, it further generalizes the approach to cross-sectional multilevel data with more than two correlated random effects, thereby permitting a more comprehensive representation of hierarchical dependencies and cluster-level variability. These methodological advancements underscore the adaptability and scalability of INLA in managing complex MBNs, thus opening new avenues for structure and parameter learning in contexts involving small sample correlated data.

Previous research has investigated BNs for related (clustered) data using MLE, but with some limitations. Scutari et al.^11^ concentrated solely on Gaussian nodes, while Azzimonti et al.^46^ focused exclusively on categorical nodes. Both studies noted a decline in MLE performance when dealing with small group sizes. Conversely, our study illustrates that MBNs fitted using INLA outperform MLE in scenarios characterized by small group sizes (for cross-sectional multilevel data) or a limited number of observations per individual (in longitudinal data settings). Significantly, this research extends beyond existing methodologies by incorporating both Gaussian and hybrid MBNs, thereby enhancing the applicability of our approach to more realistic and heterogeneous datasets. Furthermore, we investigated the application of INLA under two distinct prior formulations, namely the Wishart prior and LKJ prior, using two different prior settings to evaluate the sensitivity of both structural discovery and parameter estimation to the choice of prior. Our results suggest that both the learned network structure and parameter estimates exhibit mild sensitivity to the selected prior, underscoring the significance of prior specification in the Bayesian framework, which is more obvious in small sample scenarios. The apparent advantage that the weakly informative Wishart prior exhibits in the simulation experiments could be investigated and concretely quantified in future research.

This study reveals that MBNs learned using INLA with the LKJ and Wishart priors generally outperforms those learned using MLE in longitudinal and cross-sectional multilevel data scenarios for data with 30 individuals or groups, respectively, for Gaussian and hybrid MBNs. Nonetheless, the performance of hybrid MBNs still requires improvement in both scenarios for hierarchical data with 30 groups or individuals. Since the structure learning of hybrid MBNs is sensitive to prior specification, we recommend that future studies explore optimal prior setups and sensitivities for hybrid MBNs.

This study considered a sparse network structure with 10 nodes and an arc inclusion probability of 0.2, which yields relatively low-dimensional local random effects structures. We acknowledge that, as the number of nodes and random effects per node increases, the complexity of the variance–covariance estimation problem also increases. In such settings, the performance of the proposed approach is expected to benefit from a larger number of clusters and/or larger cluster sizes, which provide additional information for stable estimation of variance components. In more complex settings, particularly for larger networks with high-dimensional hyperparameter spaces, conducting sensitivity analyses with respect to prior specifications may be beneficial to assess the robustness of the results. Furthermore, the use of random restarts in the hill-climbing search may be beneficial to mitigate potential sensitivity to initial values.

## Supplemental Material

sj-pdf-1-smm-10.1177_09622802261468020 - Supplemental material for Advancing multilevel Bayesian networks with efficient Bayesian inference Supplemental material, sj-pdf-1-smm-10.1177_09622802261468020 for Advancing multilevel Bayesian networks with efficient Bayesian inference by Bezalem Eshetu Yirdaw, Legesse Kassa Debusho, Janet van Niekerk and Håvard Rue in Statistical Methods in Medical Research
