# Supplementary material for: Advancing multilevel Bayesian networks with efficient Bayesian inference
Source: Stat Methods Med Res. 2026 Sep 10;35(9):1910–31. doi: 10.1177/09622802261468020 (PMC13562775; doi:10.1177/09622802261468020)
Supplement: Supplementary file 1 [file sj-pdf-1-smm-10.1177_09622802261468020.pdf]

Advancing multilevel Bayesian networks with efficient Bayesian inference

Journal Title
XX(X):1–7
©The Author(s) 2025
Reprints and permission:
sagepub.co.uk/journalsPermissions.nav
DOI: 10.1177/ToBeAssigned
www.sagepub.com/
SAGE

Supplementary materials

This section presents supplementary results from the simulation study based on the true MBN 2 which was conducted to evaluate and compare the performance of structure and parameter learning of MBN under various simulation conditions discussed in the simulation study Section of the main manuscript. The Figures and Tables included here provide detailed summaries of the results, such as SHD, F1 score of the structure, classification accuracy of the grouping variables, and KLD of the parameters of true MBN 2 for Gaussian and hybrid MBN across simulation scenarios. An exploratory data analysis for the real-data application in the main manuscript is provided in the last section of the supplementary material.. These results support and complement the main findings presented in the main body of the manuscript.

Parameters learning

This section presents the parameter learning of the hybrid MBN under different simulation conditions in a cross-sectional multilevel data setting.

Table S1. Parameter learning based on the true DAG for MBN with k correlated random effects, a case of multilevel cross-sectional data with 30 groups

Child		Parent	Type of Estimate	True Parameter	MLE	I-Wishart	W-Wishart	W-LKJ	I-LKJ
Y_1	Level I		$\hat{\beta}_0$	0.30	-0.03	-0.03	-0.03	-0.03	-0.03
	Level II	G	$\hat{\sigma}^2_{u_0}$	0.35	$4.6e^{-8}$	$9.9e^{-3}$	0.09	0.07	0.26
Y_3			$\hat{\beta}_0$	0.30	0.56	0.55	0.56	0.65	0.66
	Level I	Y_1	$\hat{\beta}_2$	-0.5	-0.51	-0.49	-0.50	-0.59	-0.59
		G	$\hat{\sigma}^2_{u_0}$	0.45	0.19	0.01	0.10	0.12	0.31
	Level II	Y_1	$\hat{\sigma}^2_{u_1}$	0.35	$2.3e^{-4}$	0.01	0.11	0.19	0.17
			$\hat{\sigma}_{u_{01}}$	-0.16	$6.5e^{-3}$	$1.7e^{-7}$	$1.0e^{-3}$	0.04	0.05
Y_6			$\hat{\beta}_0$	1.03	0.74	0.62	0.70	0.65	0.65
	Level I	Y_1	$\hat{\beta}_2$	-0.27	-0.27	-0.33	-0.26	-0.25	-0.25
		Y_5	$\hat{\beta}_3$	0.77	0.80	0.84	0.81	0.83	0.83
		G	$\hat{\sigma}^2_{u_0}$	0.54	0.21	0.01	0.18	0.23	0.23
	Level II	Y_1	$\hat{\sigma}^2_{u_1}$	0.43	0.66	0.01	0.36	0.57	0.57
		Y_5	$\hat{\sigma}_{u_2}$	0.34	0.20	0.01	0.22	0.29	0.29
			$\hat{\sigma}_{u_{01}}$	0.14	0.15	$1.4e^{-4}$	0.13	0.11	0.11
			$\hat{\sigma}_{u_{02}}$	0.04	0.20	$1.0e^{-3}$	0.09	0.07	0.07
			$\hat{\sigma}_{u_{12}}$	0.11	0.16	$5.9e^{-4}$	0.14	0.15	0.15

Table S2. Parameter learning based on the true DAG for MBN with k correlated random effect, a case of multilevel cross-sectional data with 50 groups

Child		Parent	Type of Estimate	True Parameter	MLE	I-Wishart	W-Wishart	W-LKJ	I-LKJ
Y_1	Level I		$\hat{\beta}_0$	0.30	0.31	0.308	0.315	0.361	0.361
	Level II	G	$\hat{\sigma}^2_{u_0}$	0.35	0.09	0.010	0.100	0.087	0.328
Y_3			$\hat{\beta}_0$	0.30	0.505	0.506	0.509	0.544	0.544
	Level I	Y_1	$\hat{\beta}_2$	-0.5	-0.798	-0.788	-0.798	-0.864	-0.864
		G	$\hat{\sigma}^2_{u_0}$	0.45	0.107	0.010	0.105	0.141	0.274
	Level II	Y_1	$\hat{\sigma}^2_{u_1}$	0.35	0.100	0.011	0.114	0.164	0.182
			$\hat{\sigma}_{u_{01}}$	-0.16	0.104	$4.4e^{-7}$	0.007	0.020	0.069
Y_6			$\hat{\beta}_0$	1.03	1.219	1.188	1.226	1.147	1.146
	Level I	Y_1	$\hat{\beta}_2$	-0.27	-0.369	-0.361	-0.371	-0.376	-0.376
		Y_5	$\hat{\beta}_3$	0.77	0.591	0.609	0.587	0.605	0.605
		G	$\hat{\sigma}^2_{u_0}$	0.54	0.277	0.019	0.116	0.284	0.281
	Level II	Y_1	$\hat{\sigma}^2_{u_1}$	0.43	0.220	0.015	0.154	0.319	0.318
		Y_5	$\hat{\sigma}_{u_2}$	0.34	0.380	0.115	0.343	0.435	0.439
			$\hat{\sigma}_{u_{01}}$	0.14	-0.110	0.007	0.021	-0.037	-0.056
			$\hat{\sigma}_{u_{02}}$	0.04	0.134	0.032	0.059	-0.007	-0.024
			$\hat{\sigma}_{u_{12}}$	0.11	0.358	0.024	0.077	0.112	0.130

Structure learning

This section presents additional simulation results based on the true MBN 2.

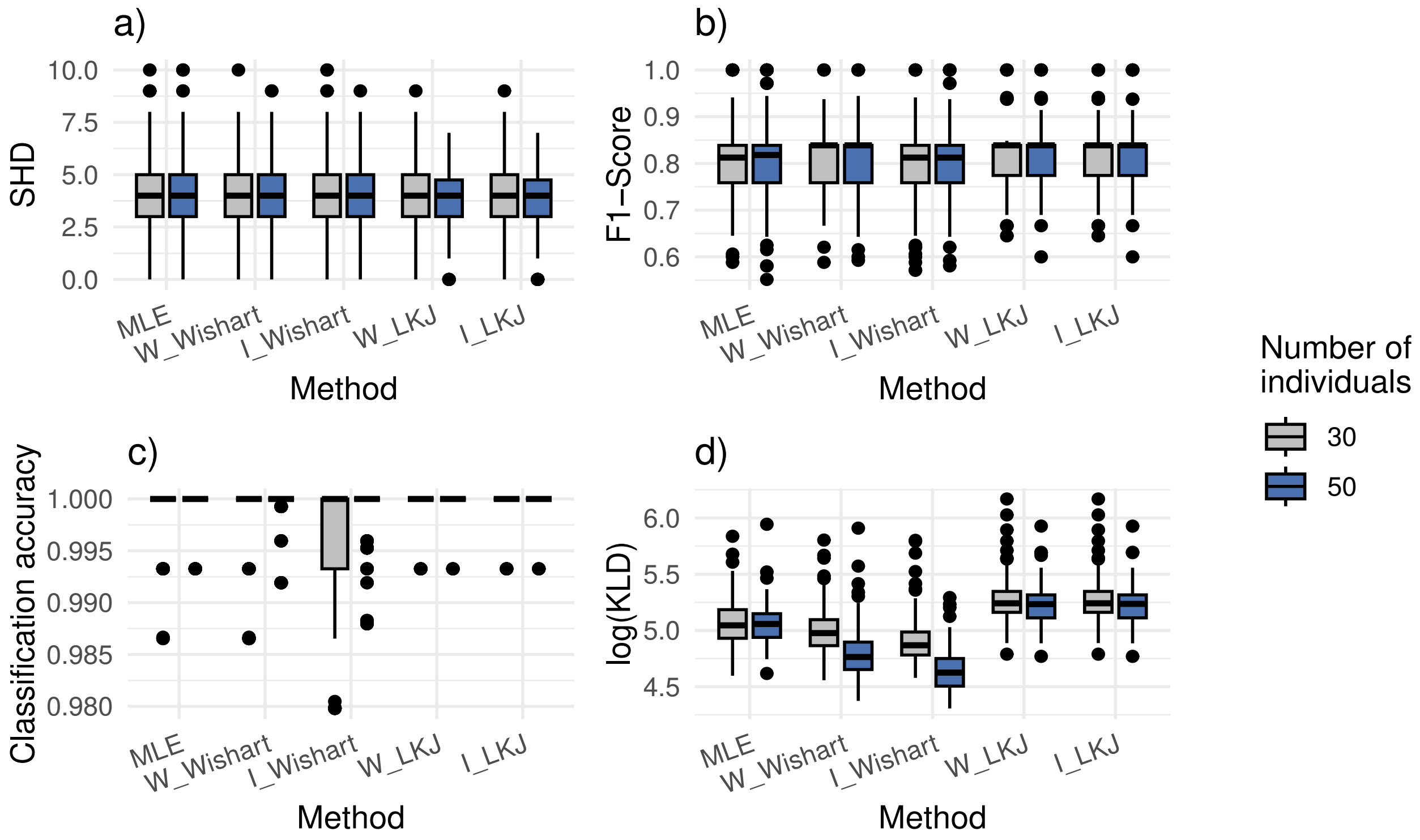

Figure S1. Performance comparison of Gaussian MBN under different estimation methods and prior choices, including MLE, weakly and more informative Wishart priors (W-Wishart, I-Wishart), and weakly and more informative LKJ priors (W-LKJ, I-LKJ), for longitudinal data with varying higher level units. Results correspond to true MBN 2

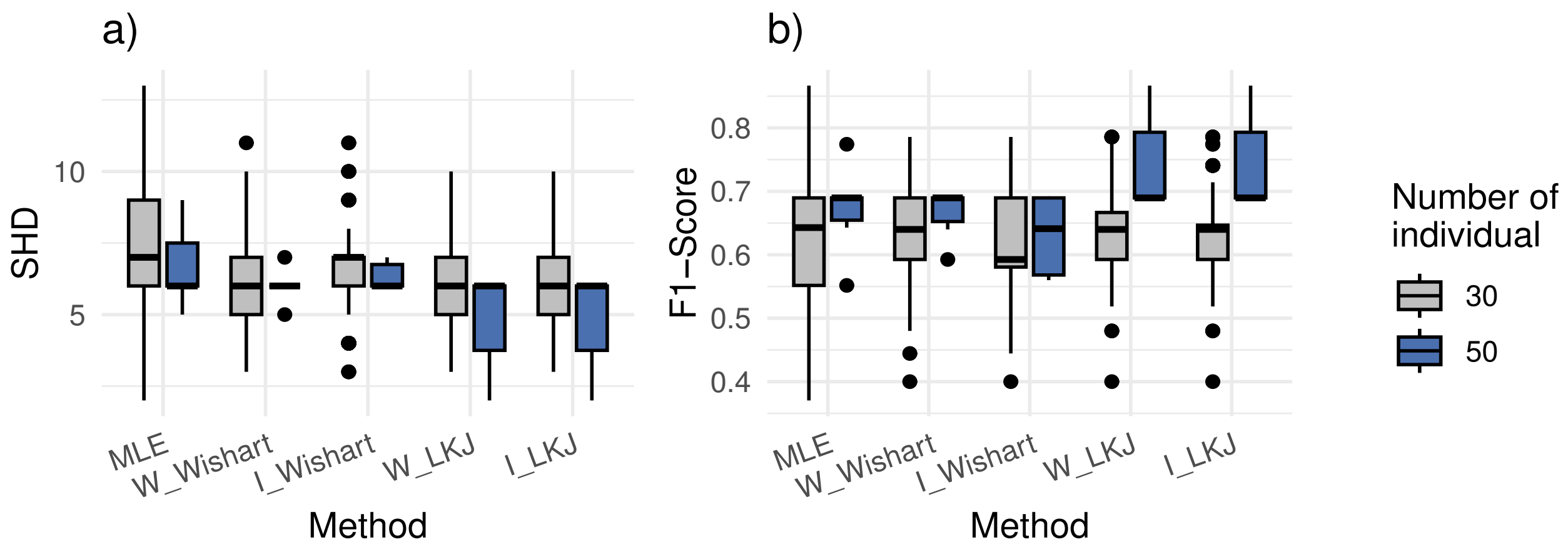

Figure S2. Performance comparison of hybrid MBN under different estimation methods and prior choices, including MLE, weakly and more informative Wishart priors (W-Wishart, I-Wishart), and weakly and more informative LKJ priors (W-LKJ, I-LKJ), for longitudinal data with varying higher level units. Results correspond to true MBN 2

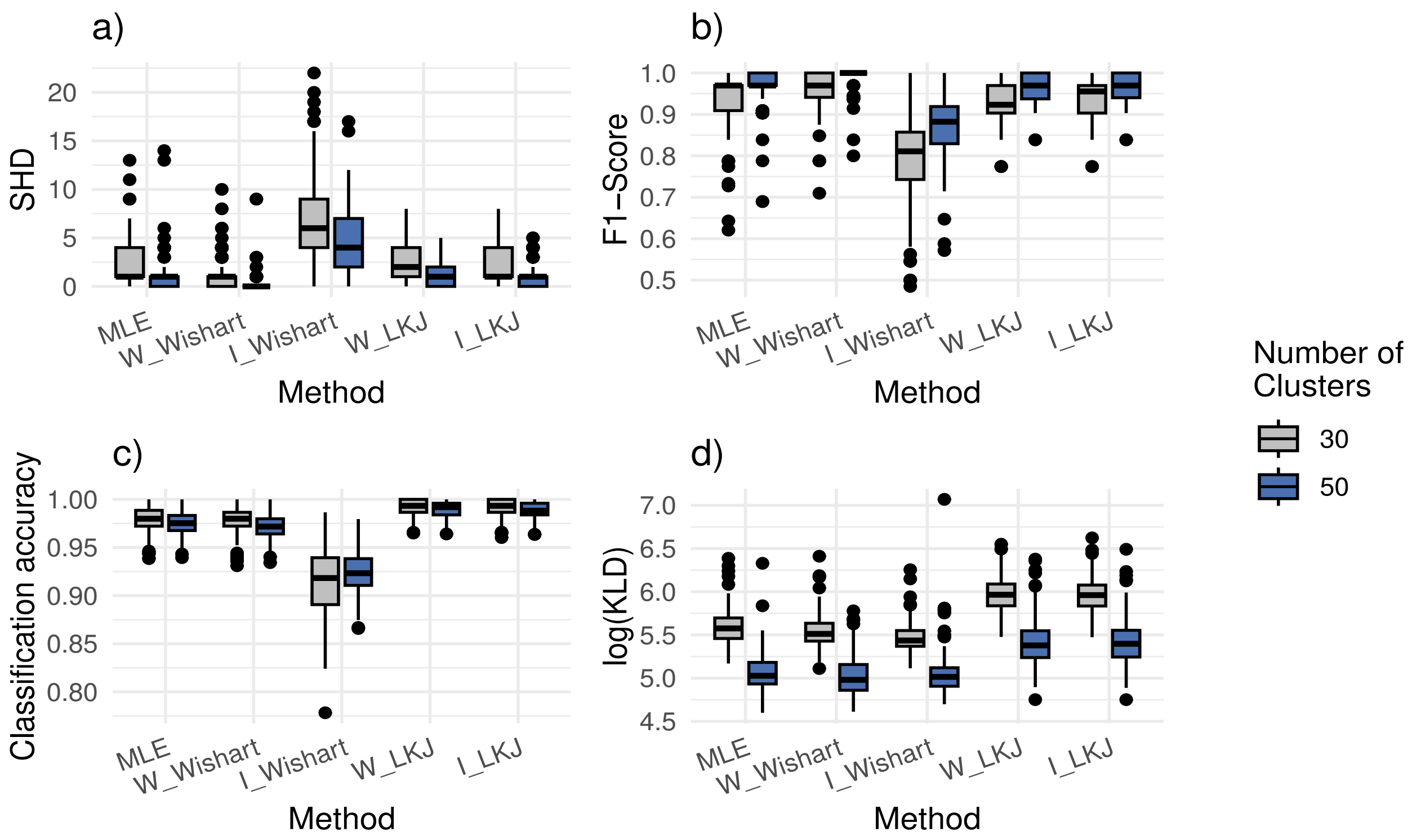

Figure S3. Performance comparison of Gaussian MBN under different estimation methods and prior choices, including MLE, weakly and more informative Wishart priors (W-Wishart, I-Wishart), and weakly and more informative LKJ priors (W-LKJ, I-LKJ), for multilevel cross-sectional data with varying higher level units. Results correspond to true MBN 2

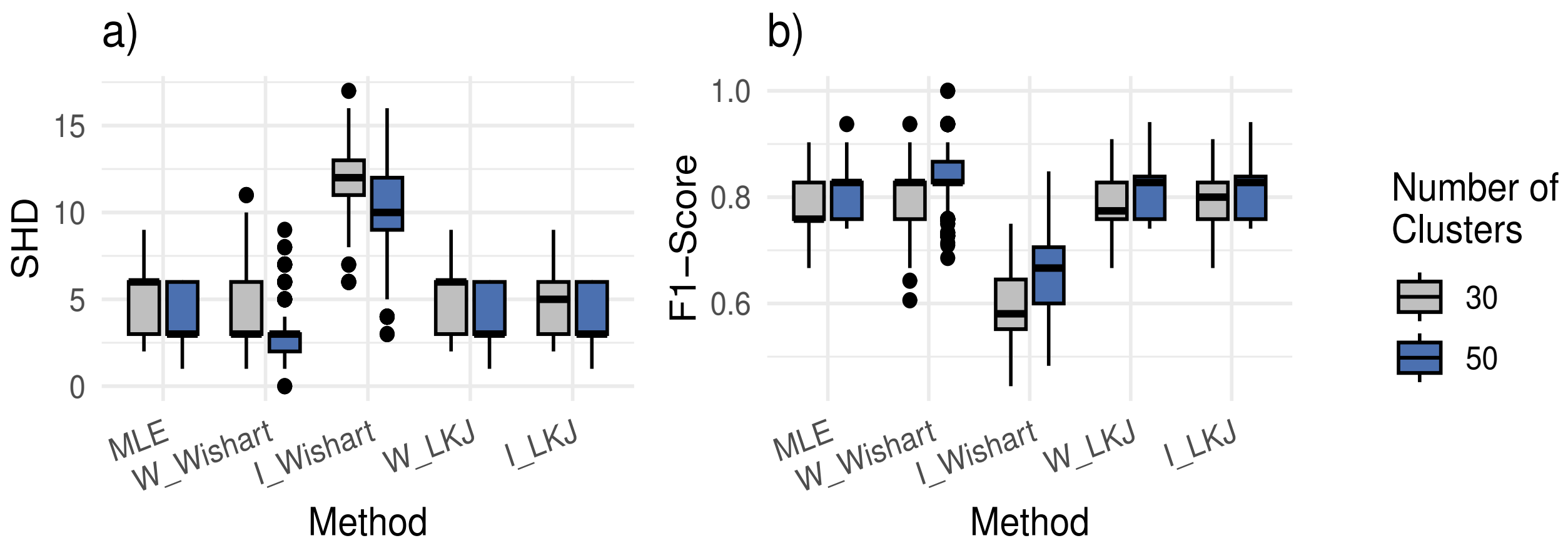

Figure S4. Performance comparison of hybrid MBN under different estimation methods and prior choices, including MLE, weakly and more informative Wishart priors (W-Wishart, I-Wishart), and weakly and more informative LKJ priors (W-LKJ, I-LKJ), for multilevel cross-sectional data with varying higher level units. Results correspond to true MBN 2

Exploratory analysis

This section presents summary statistics and an exploratory analysis of the data used in the empirical application. Table S3 summarizes the baseline summary statistics for the variables. Among 120 children, 48.33% were female, and 51.67% were male. The mean age of the children was 22.65 with a standard deviation (SD) of 14.23 at the start of the study. In addition, 26 (21.67%) of the children suffered from diarrhea at the study's baseline. Furthermore, a few children, 16.33%, 21.67%, and 20.8%, were suffering from AGE, pneumonia, and dehydration at the baseline of the study, respectively. Moreover, the mean WHZ score of children was -3.04 with SD of 1.28 at the study's baseline (Table S3).

Table S3. Summary statistics of variables at the baseline of the study.

Variable	Count	frequency/mean	Percent/SD
Gender	Female	58	48.33
	Male	62	51.67
Diarrhea	No	94	78.33
	Yes	26	21.67
AGE	No	100	83.33
	Yes	20	16.33
Pneumonia	No	95	79.16
	Yes	25	20.8
Dehydration	No	90	75.00
	Yes	30	25.00
WHZ		-3.04	1.28
Age		22.65	14.23

The caterpillar plot in Fig. S5 shows the proportion of the binary outcomes: diarrhea, dehydration, AGE, and pneumonia across children. It is observed that the proportion of diarrhea, dehydration, AGE, and pneumonia is different across children. Furthermore, the individual and mean profile plots of WHZ over time in Fig. S6 highlight a notable variation between subjects.

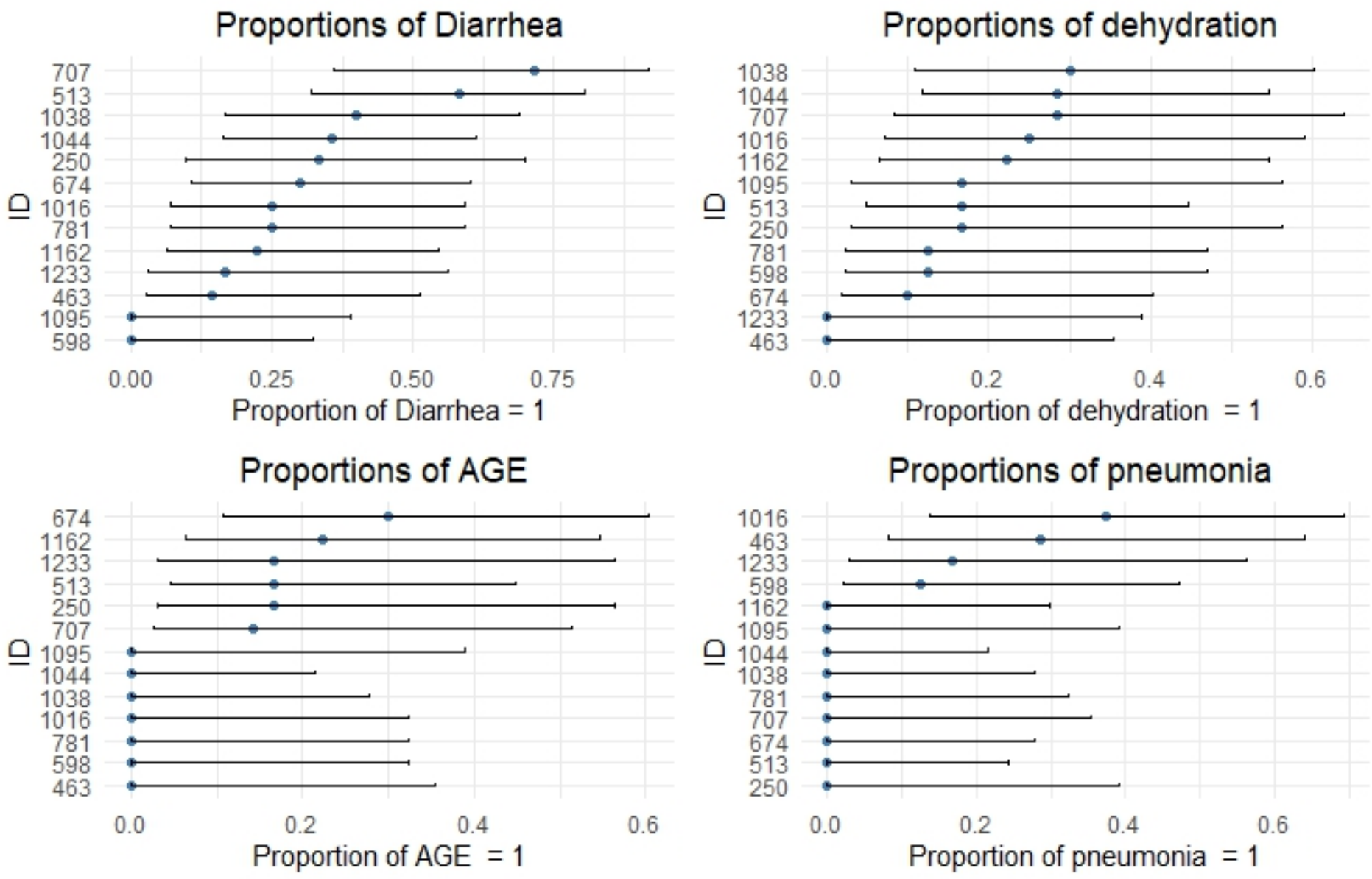

Figure S5. Caterpillar plot showing the estimated proportion of children with diarrhea, dehydration, pneumonia and AGE across individuals

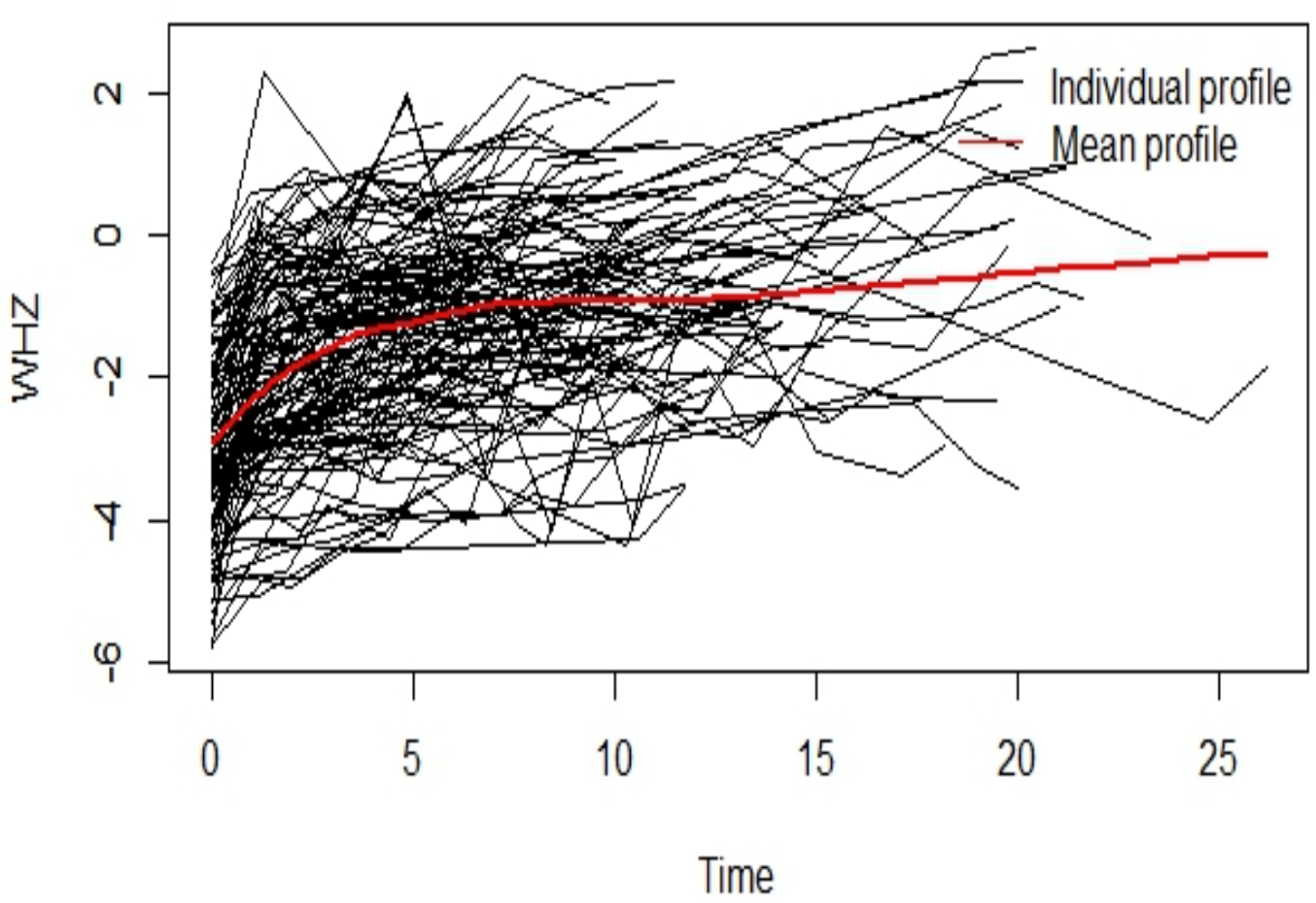

Figure S6. Individual and mean profile plot for WHZ

Sample r code

A sample R code snippet for the score function of the informative INLA approach in the longitudinal data setting for Gaussian MBN is provided below. The complete implementation is available in the Figshare repository referenced in the manuscript.

```
fitME1 = function(node, parents, group, t,
    data) {
  nid <- length(unique(data[[group]]))
  data$numid <- as.numeric(as.factor(data[[
      group]]))
  data$slopeid <- data$numid +nid

  rhs = paste(c("1", setdiff(parents, t), t)
      , collapse = " + ")

  formula = as.formula(paste0(node, "~", rhs
      ,
                              "+ f(numid,
                                 model = '
                                 iidkd', n =
                                 ", 2 * nid,
                                  ", order =
                                  2)",
                              "+ f(slopeid,
                                 t, copy = '
                                 numid')"))

  model = tryCatch(
    inla(formula, family = "gaussian", data
        = data,
         control.inla = list(int.strategy =
             "eb")),
    error = function(e) {
      message("Error fitting model for node
          ", node, ": ", e$message)
      return(NULL)
    }
  )
  return(model)
}

scoreME1 =  function(node, parents, data,
    args = list()) {

  if (node == args$group)
    return(0)
  if (node == args$t)
    return(0)

  require(INLA)

  local.distribution =
    fitME1(node = node, parents = setdiff(
        parents, c(args$group, args$t)),
           group = args$group, t = args$t,
               data = data)

  if (is.null(local.distribution))
    return(-Inf)

  return(local.distribution$mlik[1])


}
```
